# Modelling kidney cystogenesis using human kidney tubuloid cultures

**DOI:** 10.1186/s12860-026-00591-x

**Published:** 2026-05-11

**Authors:** Jana Rohe, Arsila Palliyulla Kariat Ashraf, Melina Kehl, Xenia Thielmann, Jörg Ellinger, Glen Kristiansen, Dagmar Wachten, Marieta Ioana Toma

**Affiliations:** 1 Institute of Pathology, University Hospital Bonn (UKB), Medical Faculty, University of Bonn, Venusberg-Campus 1, Building 62, 53127 Bonn, Germany; 2 Institute of Innate Immunity, Biophysical Imaging, University of Bonn, University Hospital, BMZ-II, Venusberg- Campus 1, 53127 Bonn, Germany; 3 Department of Urology and Pediatric Urology, University of Bonn, University Hospital Bonn, Bonn, Germany

**Keywords:** ADPKD, cAMP signalling, Cilia, Dome tubuloids, Organoids, Kidney cyst, Kidney epithelial cells, Suspension tubuloids

## Abstract

**Background:**

Renal cyst formation, as observed in autosomal dominant polycystic kidney disease (ADPKD), is a life-threatening condition with no effective cure yet. The molecular mechanisms underlying primary cilia dysfunction, which causes cyst formation and disease development, are not well understood. Human kidney tubuloids offer a promising model system to investigate the disease mechanisms of PKD in physiologically relevant 3D structures. However, their inherent cystic morphology poses a challenge in effectively modelling kidney cystogenesis. Therefore, our study aims to refine the culture method of tubuloids and assess the efficacy of these modified cultures in modeling cyst formation and development.

**Results:**

We developed human kidney tubuloid models derived from adult kidney tubular cells using different methods for 3D in-vitro cultures. Tubuloids cultured in suspension or an extracellular matrix scaffold manifested distinctly polarized epithelial structures. Bulk RNA sequencing and immunohistochemistry revealed differential transcriptional profiles, highlighting variations in cellular composition and cellular fate within the kidney epithelium between the two types of tubuloids. Notably, the experimental activation of chronic cAMP signalling promoted cyst formation in vitro, validating the suitability of these tubuloids for studying kidney cystogenesis. Furthermore, we demonstrate that tubuloids are amenable to genetic modification through recombinant adeno-associated virus transduction.

**Conclusions:**

Our study identifies different in-vitro tubuloid cultures as relevant model systems for examining the molecular and cellular changes involved in kidney cystogenesis in humans. These models will enhance our capability to discover novel pathogenetic mechanisms underlying ADPKD and validate candidate drugs for clinical application.

**Supplementary Information:**

The online version contains supplementary material available at 10.1186/s12860-026-00591-x.

## Graphical Abstract

**Supplementary Information:**

The online version contains supplementary material available at 10.1186/s12860-026-00591-x.

## Graphical abstract

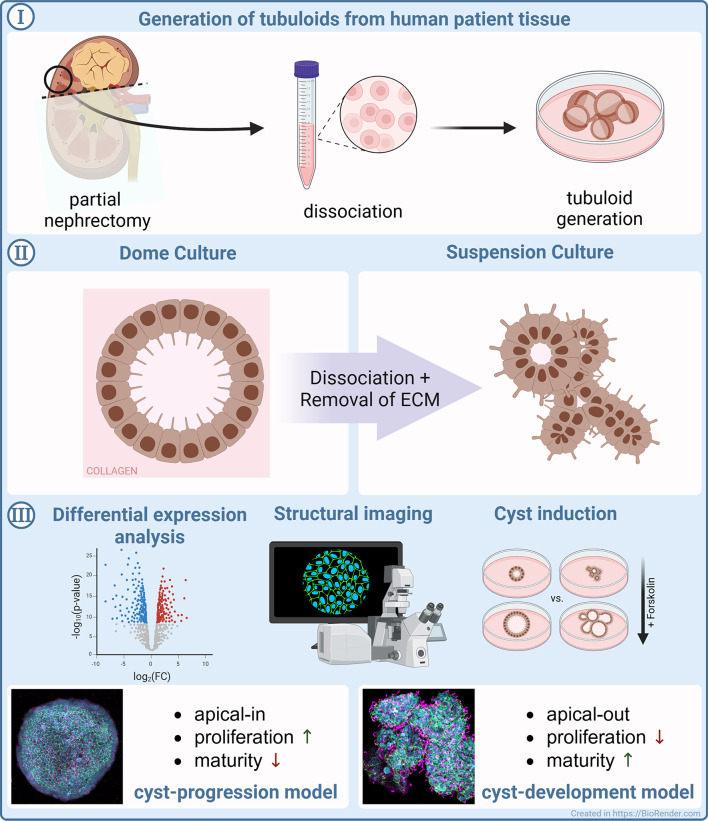

## Background

Polycystic kidney disease (PKD) is an inherited disorder characterized by kidney enlargement due to the formation of fluid-filled cysts. Kidney cysts originate from the epithelium of the kidney tubule and progressively disrupt surrounding tissue, impairing kidney function and resulting in kidney failure [1, 2]. Dysfunction of the primary cilium, a microtubule-based protrusion of the plasma membrane, underlies cyst formation in PKD [3, 4]. The primary cilium functions as a cellular antenna, sensing extracellular cues and translating these signals into intracellular responses, thereby regulating cellular functions such as proliferation and differentiation [5, 6]. Primary cilia dysfunction leads to severe disorders called ciliopathies, with PKD being a prominent example.

The molecular mechanisms underlying renal cyst formation have been extensively studied for Autosomal Dominant PKD (ADPKD), which is the most prevalent monogenic kidney disorder (1:1000) [7], and is caused by disease-causing variants in *PKD1* and/or *PKD2*, encoding polycystin-1 (PC1) and polycystin-2 (PC2), respectively [8, 9]. Together, PC1/PC2 form a mechanosensitive protein complex in primary cilia of kidney epithelial cells, where they conduct Ca^2+^ in response to fluid flow in the kidney tubules [10]. Of note, recent evidence suggests that PC1 and PC2 are also found in non-ciliary localizations [11], indicating that PC dysfunction outside the cilium might also need to be considered for ADPKD development. In ADPKD, PC1/2 dysfunction impairs ciliary Ca^2+^ signalling, which has been proposed to increase ciliary cAMP levels by relieving the Ca^2+^-dependent inhibition of adenylyl cyclase (AC) AC5 and AC6 [12, 13]. Furthermore, cAMP levels are elevated in kidney cysts in vivo [14], and an increase in intracellular cAMP levels in kidney epithelial cells in vitro leads to cyst formation [15]. Shedding light on the cAMP-dependent mechanisms underlying cyst formation, we have applied spatial optogenetics to manipulate ciliary cAMP signalling and identified a novel cAMP signalosome in the primary cilia of kidney epithelial cells that drives cystogenesis [16]. It relies on the cAMP-dependent activation of Protein Kinase A (PKA) in the cilium, which phosphorylates the transcription factor CREB (cyclic AMP-Responsive Element Binding-Protein). In turn, ciliary-activated pCREB drives a gene expression program distinct from the gene expression program evoked by cAMP in the cytoplasm, leading to cyst formation [16]. To investigate whether these disease-causing mechanisms also underly cystogenesis in human renal epithelial cells, better models are needed.

In recent years, gene-edited human pluripotent stem cell (hPSC)-derived kidney organoids [17–22], induced PSCs (iPSCs)-derived kidney organoids from PKD patients [23, 24], and human adult stem cell (ASC)-derived kidney organoids [25, 26] have been established to model PKD, reflecting the human in-vivo situation. The widely studied iPSC-derived in-vitro kidney organoid models of PKD have the advantage of structural complexity and differentiate into mini-kidney structures; however, at the expense of taking several months to develop into tissue that does not resemble adult kidney tissue [27]. In contrast, ASC-derived kidney organoids, or ‘tubuloids’, are a fast, simplistic, and genetically stable model system [28]. Tubuloids contain differentiated, functional epithelial cells, exclusively representing the kidney epithelium, and appear as highly polarized epithelial structures [26, 28]. Thus, tubuloids are restricted to model function and disease of the epithelial elements of the kidney, which is relevant in the context of PKD, as kidney cyst formation and development are associated with disruption of epithelial homeostasis.

Although tubuloids are an efficient model system, they intrinsically display typical cystic morphology [28], complicating the discernment of induced or developed cysts under disease conditions and limiting their utility for studying kidney cystogenesis. It is unclear why these organoids invariably appear as cystic structures. To overcome this challenge, we aimed to adapt the culture method of tubuloids to optimize their efficiency and utility in ADPKD research.

Here, we generated human kidney tubuloids derived from adult kidney tubular cells using different three-dimensional (3D) in-vitro cultures. By comparing tubuloids in suspension and in extracellular matrix (ECM) scaffold, we identified distinctly polarized epithelial structures that demonstrated varied cellular compositions and fates. Furthermore, we have combined pharmacology and tissue imaging to demonstrate that these tubuloids develop cysts in vitro upon chronic stimulation of cAMP signalling and present functional read-outs to quantify kidney cysts in tubuloids. Thereby, we identify different tubuloid models as valuable tools to study cAMP-dependent kidney cyst development and progression, which will shed light on the pathomechanisms underlying PKD.

## Methods

### Preparation of L-WRN conditioned medium

Murine L-WRN cell line (ATCC, #CRL-3276) was used as a source for producing Wnt-3a (W), R-spondin 3 (R), and noggin (N) conditioned medium. The L-WRN conditioned medium was prepared according to the ATCC’s protocol.

### Human kidney cell isolation

Fresh adult human nephrectomy or partial nephrectomy tissues were obtained from the University Hospital Bonn (UKB; Biobank Bonn). The study includes samples from seven donors with the following diagnoses: three with clear cell renal cell carcinoma (ccRCC), two with papillary RCC, one with oncocytoma, and one with chromophobe renal cell carcinoma. Tumor-adjacent non-neoplastic kidney tissue was isolated, minced, and dissociated using gentleMACS™ Octo Dissociator and the Tumor Dissociation Kit, human (Miltenyi Biotec, #130-095-929). The dissociated cells were passed through a 100 μm strainer (Corning, #431752), resuspended in the collection medium containing Advanced DMEM/F-12 medium (Gibco, #12634028) supplemented with 1x GlutaMAX (Gibco, #35050061), 1 M HEPES (Roth, #9157.1), 100 µg/ml Normocin (InvivoGen, #ANT-NR-1) and 2.5 µg/ml Amphotericin B (Thermo Fisher Scientific, #15290018), and subjected to centrifugation for 5 min at 500 x g and 4 °C. The supernatant was removed, the pellet was resuspended in red blood cell lysis buffer (Sigma, #R7757), and incubated on ice for 3 min. The reaction was stopped by adding PBS, followed by centrifugation for 5 min at 500 x g and 4 °C. The supernatant was removed, and the pellet was resuspended in the collection medium. Afterwards, the single-cell suspension was subjected to tubuloid culture.

### Tubuloid cultures

Tubuloid cultures were maintained in organoid complete medium containing basic organoid medium (see supplementary information, Tab. S9) and L-WRN conditioned medium in a 1:1 ratio, supplemented with 10 µg/ml Normocin (InvivoGen, #ANT-NR-1), 10 µM Y-27,623 (Biomol, #AG-CR1-3564-M010), 50 ng/ml hEGF (Sigma, #SRP3027), 1.2 µM SB 202,190 (BioGems, #1523077) and 0.5 µM A83-01 (Sigma, #SML0788), at 37 °C under 5% CO_2_.

To generate dome tubuloids, dissociated single cells were resuspended in an ice-cold collagen solution containing Cellmatrix Type I-A collagen (Nitta Gelatin Inc., #637–00653), 10 x Ham’s F12 Nutrient Mix (Gibco, #21700018), and reconstitution buffer (see supplementary information, Tab. S13) in an 8:1:1 ratio. Small domes of collagen-cell suspension were placed into a 6-well multi-well plate and incubated upside-down for 20 min at 37 °C under 5% CO_2_. After the domes polymerized, they were covered with the organoid complete medium. The medium was changed every 3–4 days. Dome tubuloids were passaged before they became too large or necrotic, typically between 7 and 14 days post-seeding. The collagen scaffold was digested using 1 mg/ml collagenase IV (Rockland, #MB-121-0100) in the collection medium, incubated for 30 min at 37 °C under 5% CO_2_, and harvested by centrifugation for 5 min at 300 x g. The supernatant was removed, and the pellet was resuspended in 0.25% Trypsin-EDTA (Thermo Fisher Scientific, #25200056) and incubated for 10 min at 37 °C under 5% CO_2_. The cell suspension was neutralized with the collection medium, and dissociated tubuloids were collected by centrifugation for 5 min at 300 x g and 4 °C. The supernatant was removed, and the cells were reseeded in collagen domes or suspension culture.

To generate tubuloids in suspension, dissociated cells from dome tubuloids were seeded in 24-well multi-well ultra-low attachment (ULA) plates (Thermo Fisher Scientific, #174930) and cultured in the organoid complete medium under static conditions. Media exchange was performed by carefully collecting the cell suspension from the plate and followed by centrifugation for 3 min at 300 x g. The supernatant was removed, and the pellet was resuspended gently in the organoid complete medium and reseeded into the plate.

### Histology and immunohistochemistry

Tubuloids were fixed overnight in 4% stabilized formaldehyde solution (Sigma-Aldrich, #252549) at 4 °C. The next day, tubuloids were resuspended in liquified HistoGel (Epredia, #12006679) by heating to 60 °C and filled into cryomolds. After solidifying for 20 min at 4 °C, the HistoGel with the specimen was removed and placed into tissue cassettes. Tissues were dehydrated and cleared in a clearing agent and Xylene using the Sakura, Tissue-Tek^®^ VIP^®^ 6 AI Vacuum Infiltration Processor before incubating in molten paraffin wax (HistoComp Gewebeeinbettmittel, Vogel MedTec, #Vo-5-1001). Tissues were cast into molds with liquid paraffin and cooled to form a solid paraffin block with embedded tissue using the Medite TES Valida^®^ Paraffin Embedding Station. Paraffin-embedded tubuloids were sliced into 2 μm sections using a pfm medical Rotary 3006 EM Microtome and mounted on TOMO^®^ adhesive glass slides (Matsunami, #TOM-11). Following, slides were dried for 30 min at 65 °C. For histological analysis, tubuloid sections were stained with Haemalaum acid (Waldeck GmbH & Co. KG, #2E-038) and Eosin (Eosin solution 1%, Waldeck GmbH & Co. KG, #2 C-289) (H&E) using the Medite TST 44.000 Multistainer combined with the Leica HistoCore SPECTRA CV Coverslipper.

Immunohistochemistry was performed using Lab Vision™ Autostainer 480 S-2D slide stainer in accordance with the manufacturer’s instructions. The dried slides were subjected to heat-induced antigen retrieval in PT module citrate buffer 10X pH 6.0 (Vitro Master Diagnóstica, #MAD-004071R/D) at 99 °C for 20 min. Next, slides were washed in wash buffer (medac diagnostika, #B1-30AW) and distilled water, incubated with primary antibodies, and subjected to blocking using Peroxide Block (ScyTek, #ACA999). For immunodetection, BrightVision+ Anti-IgG Polymer (Poly-HRP, ImmunoLogic, #C-DPVB999HRP) and Histofine DAB-3 S Kit (Nichirei Biosciences, #495192F) were added. Counterstaining with Haematoxylin was performed manually. Following, slides were dehydrated and mounted with PERTEX^®^ mounting medium (Leica, #LEIC801) using the Leica HistoCore SPECTRA CV Coverslipper.

For cadherin-16 (CDH16) staining, slides were deparaffinized using EZ Prep concentrate (Ventana, #950 − 102) and pretreated with target retrieval solution Ultra CC1 (Ventana, #950 − 224). Staining was performed using an automated staining system Roche BenchMark Ultra Autostainer in accordance with the manufacturer’s instructions. Immunodetection was performed with the UltraView Detection Kit (Roche, #760 − 500). GATA3 was detected using the OptiView detection kit and Amplifier (Roche, #06396500001, #06396518001).

Embedding, slicing, and staining were conducted by the routine laboratory of the Institute of Pathology at the University Hospital Bonn (UKB).

Antibodies used for immunohistochemistry are described here (see supplementary information, Tab. S7).

### Cyst induction

Dissociated cells from dome and suspension tubuloids at passage P2 were seeded in uncoated flat-bottom and ULA 96-well multi-well plates, respectively, at a density of 1 × 10^4^ cells per well and cultured in the organoid complete medium. After seven days of culture, tubuloids were incubated with the organoid complete medium supplemented with either 10 µM Forskolin (BioGems, #66575-29-9) or DMSO (Roth, #A994.1) as vehicle control. Bright-field images were captured 5-, 24-, 48- and 72-hours post-treatment to track the cyst formation. Three days post-treatment, tubuloids were fixed overnight in 4% stabilized formaldehyde solution (VWR International, #50-00-0) at 4°C and subjected to immunofluorescence staining and microscopy.

For live-cell analysis of cyst formation in suspension tubuloids, dissociated single cells were incubated with 2 µM Calcein-AM (Biolegend, #425201) for 30 min at 37 °C under 5% CO_2_. Following, cells were seeded in a ULA 96-well multi-well plate at a density of 5000 cells per well in the organoid complete medium and cultured using the Sartorius Incucyte^®^ S3 Live-Cell Analysis System. After four days of culture, tubuloids were incubated with the organoid complete medium supplemented with either 10 µM Forskolin or DMSO for up to three days. Images were captured every three hours to track the cyst formation.

### Production of adeno-associated viruses

Recombinant adeno-associated viruses (rAAV) were produced in the Virus Core Facility at the University of Bonn, by triple calcium-phosphate mediated transfection of HEK-293T cells with the expression plasmids pAAV-CMV-PI-EGFP-WPRE-bGH (gift from James M. Wilson: Addgene plasmid #105530; http://n2t.net/addgene:105530; RRID: Addgene_105530), the rep/cap encoding plasmids pRV1 [29], and pAAV2/8 (gift from James M. Wilson: Addgene plasmid #112864), and the adenoviral helper plasmid pAdDeltaF6 (gift from James M. Wilson: Addgene plasmid #112867). For heparin column-based purification of the chimeric rAAV2/8-CMV-EGFP virus, cells were harvested 48 h post-transfection and the virus was purified using HiTrap^®^ Heparin HP affinity columns (Cytiva, #17040703) [29]. rAAVs were concentrated using Amicon^®^ Ultra Centrifugal Filter (100 kDa MWCO, Merck Millipore, #UFC5100) and sterilized through a 0.22 μm filter. The genomic titer was determined by qPCR using primers specific for EGFP.

### Tubuloid transduction

Dissociated single cells (5000 cells) from dome or suspension tubuloids at P2 were incubated with 25 µl organoid complete medium containing 10^7^ genome copies/ml of rAAV2/8-CMV-EGFP viral particles or virus storage buffer (PBS + 0.001% Pluronic F-68) for 30 min at 37 °C under 5% CO_2_. After centrifugation for 5 min at 500 x g and 4 °C, the supernatant was saved. The cell pellet was resuspended in an ice-cold collagen solution and small domes of collagen-cell suspension were placed into an uncoated flat-bottom 96-well multi-well plate which were incubated upside-down for 20 min at 37 °C under 5% CO_2_. The previously saved supernatants were added to the respective wells. The virus was diluted by adding one volume of medium 24- and 48-hours post-transduction. Transduced tubuloids were cultured for up to seven days.

### RNA extraction and library preparation bulk RNA-seq

Dome and suspension tubuloids derived from kidney tissue samples from four donors and cultured up to passages P3, P4, and P5 were harvested for bulk RNA-seq. RNA extraction from tubuloids was performed using the RNeasy Mini Kit (Qiagen, #74104) according to the manufacturer’s instructions. The RNA concentration and quality of the samples were determined with a Peqlab NanoDrop^®^ 1000 Spectrophotometer.

Library preparation was performed after RNA extraction by using the QuantSeq FWD 3´-mRNA-Seq Kit (Lexogen, #191.96) in the Next Generation Sequencing Core Facility at the University Bonn. The sequencing was executed on the Illumina NovaSeq 6000 with a NovaSeq S1 flow cell and a read length of 1 × 100 bp.

### Bulk RNA-seq processing

Bulk RNA-seq data was processed and analyzed by the Core Unit for Bioinformatics Data Analysis at the University of Bonn. The nf-core rnaseq pipeline-3.18.0 [30] was applied for the pre-processing and the quantification of the reads using the human reference genome GRCh38 and default parameters besides additional trimming for polyA and polyG. The differential expression analysis was performed using the Bioconductor package limma [31]. Statistical contrasts were calculated between the corresponding conditions defined by culture conditions and passages and the contrast defined by the average of the three contrasts between the culture conditions for each passage. To address the correlation among samples originating from the same donor, donor was considered as a random effect in the statistical model and passed to the function duplicate Correlation as blocking factor. In addition, the two experimental batches were modelled as fixed effects to correct for unwanted variation. Only genes with a minimum count of 20 in at least 3 samples were used. The Benjamini-Hochberg method was applied to calculate multiple testing-adjusted p-values for each statistical contrast. For the gene set analysis, flat (unranked) Gene Set Enrichment Analysis (GSEA) was performed using GSEA-4.3.3 (https://www.gsea-msigdb.org/) and the Hallmark and Wikipathways gene sets from the Molecular Signature Database (MSigDB) [32, 33] to evaluate the significantly enriched pathways (see supplementary information, Tab. S4 and Tab. S5, respectively). Enriched gene sets with an FDR value < 0.25 were considered significant. The heatmaps were generated with GraphPad Prism by plotting the relative expression level after normalization (log_CPM_) for individual samples. The processed and annotated data were downloaded from the CZ CELLxGENE Discover [34]. The bulk RNA-seq datasets are available at the European Genome-Phenome Archive (EGA) repository, https://ega-archive.org/datasets/EGAD50000002335. 

GO term enrichment was obtained from STRING (https://string-db.org) [35] network analysis in CytoScape-3.10.3 [36] using the stringApp-v2.2.0 [37]. All significant genes from the average differential expression of all suspension versus dome tubuloids were ranked after fold change (log_FC_) (see supplementary information, Tab. S3). The top 200 genes with the highest and lowest fold change were imported into CytoScape and plotted with a confidence cutoff of 0.7 as different STRING networks. For functional GO pathway enrichment, GO Biological Process gene sets were considered after removing redundant terms at a cutoff of 0.7.

### Immunofluorescence staining

All the following steps were performed at room temperature if not stated otherwise. Tubuloids were fixed overnight in 4% paraformaldehyde (Thermo Fisher Scientific, #43368) in PBS at 4 °C. Fixed dome tubuloids were removed from their wells and transferred into a 96-well multi-well plate. Suspension tubuloids were carefully collected in a 96-well multi-well plate after each step by centrifugation for 5 min at 300 x g and followed by removal of the supernatant. Tubuloids were washed thrice in 1% Triton X-100 (Sigma Aldrich, #X100) in PBS for 10 min each. Next, tubuloids were blocked with 1% Triton X-100 and 10% FCS in PBS overnight at 4 °C and subsequently incubated overnight with primary antibodies in 1% Triton X-100 and 10% FCS in PBS at 4 °C on a rocking shaker. Afterwards, tubuloids were washed thrice with 1% Triton X-100 and 10% FCS in PBS for 20 min each and 1% Triton X-100 in PBS for 10 min. Tubuloids were incubated with secondary antibodies and DAPI (1:10,000) (Thermo Fisher Scientific, #D1306) in 1% Triton X-100 and 10% FCS in PBS overnight at 4 °C on a rocking shaker. After washing thrice with 1% Triton X-100 in PBS for 10 min each and once with PBS, tubuloids were transferred onto a cavity glass microscopy slide and mounted with Aqua-Poly/Mount (Polysciences, #18606-20) and microscope coverslips. Object slides were dried overnight. Antibodies used for immunofluorescence are described here (see supplementary information, Tab. S8).

To capture the large, fully-inflated cysts in suspension tubuloids, untreated and Forskolin-treated tubuloids were gently isolated by removing the medium with a pipette. Afterwards, they were embedded in an ice-cold collagen solution containing Cellmatrix Type I-A collagen (Nitta Gelatin Inc., #637–00653), 10x Ham’s F12 Nutrient Mix (Gibco, #21700018), and reconstitution buffer (see supplementary information) in an 8:1:1 ratio. The mixture was dispensed as droplets onto a PhenoPlate 96-well multi-well plate (Revvity, #6055302). Once the collagen containing the suspension tubuloids polymerized in the PhenoPlate, they were fixed, stained, and imaged as described above.

### Microscopy and image analysis

Bright-field images of tubuloids were captured on the Nikon Microscope connected to the Digital Sight DS-U3 Camera Control Unit with the 5x objective. Cyst induction in suspension tubuloids was tracked live by capturing phase-contrast and fluorescent images using the Sartorius Incucyte^®^ S3 Live-Cell Analysis System with the 4x objective. Images were analyzed using QuPath-0.4.4. For dome tubuloids from each experimental condition, eight random tubuloids per well were selected, tracked, and measured at different time points. For tubuloids in suspension culture, all cysts in each well were counted and measured with the brush tool (see supplementary information, Tab. S6).

Images of H&E- and immunostained tubuloid sections were taken on the Leica DM IRB inverted research microscope and Keyence BZ-X microscope in the Institute of Pathology at the University Hospital Bonn (UKB) with the 10x and 40x objective, respectively.

Z-stacks (1 μm step Z-step size) of fluorescently stained tubuloids were taken on the Leica Stellaris 8 confocal microscope in the Microscopy Core Facility of the Medical Faculty at the University of Bonn with 40x multi-immersion objective with applied glycerol immersion.

Immunofluorescence and immunohistochemistry images were analyzed using ImageJ [38]. To obtain the width of the dome tubuloid wall, the sharpest plane among the Z-stacks was extracted using the Extract sharpest plane plugin. The wall thickness of all the dome tubuloids in the sharpest frame was measured using the line tool. The smallest wall thickness was analyzed for each tubuloid to avoid bias. The length of primary cilia in dome and suspension tubuloids was quantified using the CiliaQ plugin [39].

MIB-1 and GATA3 staining was quantified (see supplementary information, Tab. S1 and Tab. S2, respectively) by semi-automatically counting positive and negative nuclei in 40x bright field images of tubuloids using QuPath-0.4.4, with a threshold cut-off of 0.15 and Gaussian noise reduction (σ = 2 px), in combination with the Cell Counter feature for accurate classification.

### Statistical analysis

For plotting graphs and statistical analysis GraphPad Prism-10.4.0 (527) software was used. Statistical analyses used and P-values are as indicated.

## Results

### Establishment of 3D in-vitro tubuloid cultures

**Fig. 1:**
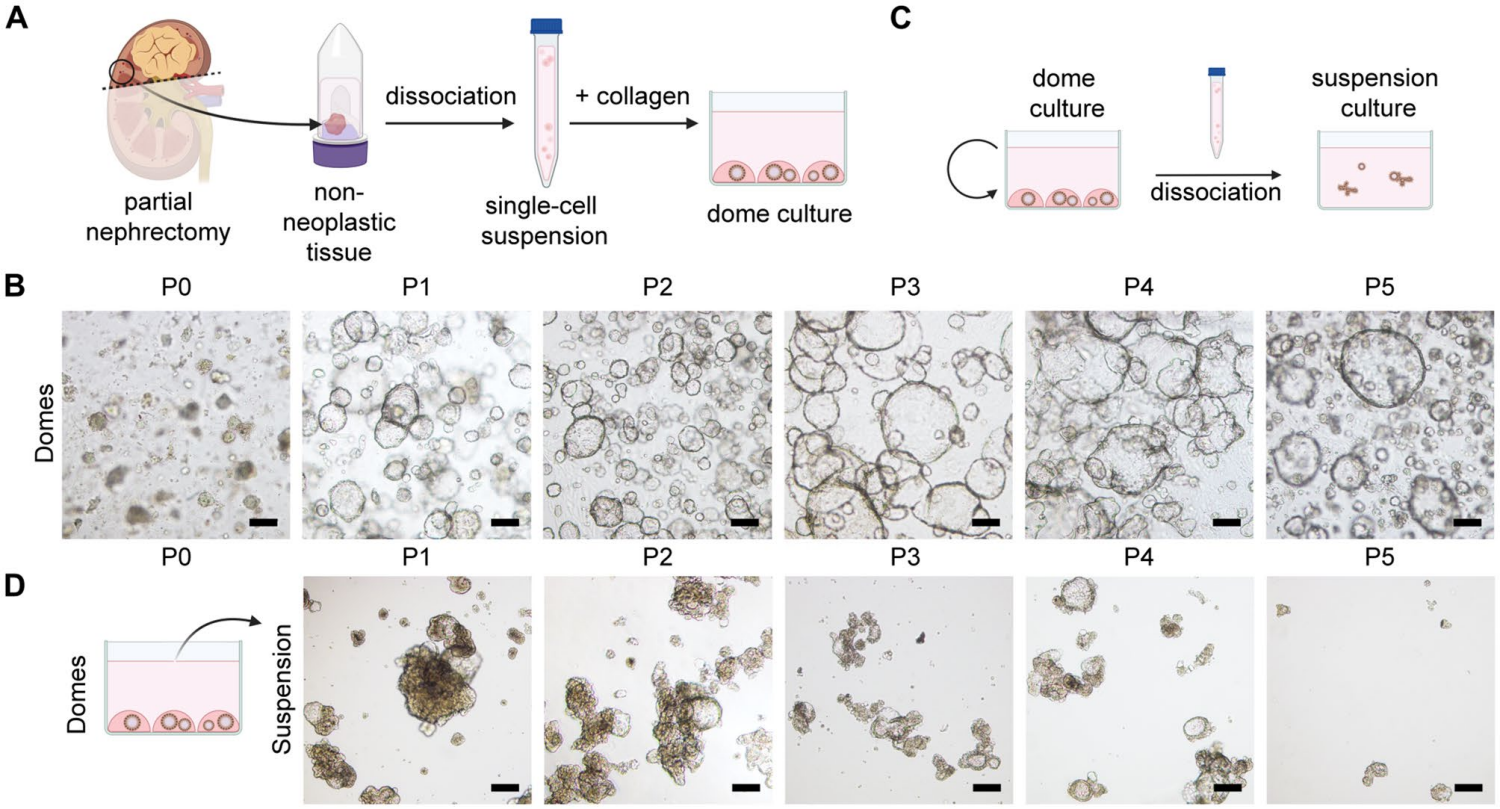
3D culture methods for human ASC-derived kidney organoids. **(A)** Schematic workflow for primary kidney cell isolation from fresh adult human nephrectomy tissues and culture of organoids in collagen domes. **(B)** Representative bright-field images of tubuloids cultured in collagen domes at different passages (P), from P0 to P5. Scale bars: 100 μm. **(C)** Schematic illustration generation suspension culture derived from dome tubuloids. **(D)** Representative bright-field images of tubuloids cultured in suspension P1 to P5. Scale bars: 100 μm. Illustrations created using BioRender

To establish tubuloids, we used adjacent non-neoplastic kidney tissue from patient specimens who underwent surgery for kidney tumors. The one-step protocol involves enzymatic and mechanical dissociation of tissue specimen into a single-cell suspension, followed by embedding into collagen domes (Fig. 1A). The dome-cultured tubuloids developed efficiently in vitro, exhibiting a stable morphology for at least five passages (Fig. 1B). In general, dome tubuloids could be maintained in culture for 90 days (Fig. S1A). As expected, these tubuloids were spherical with a typical cystic morphology characterized by a fluid-filled lumen [26, 28], which was retained throughout their development at different passages (Fig. 1B; Fig. S1A).

The extracellular gel matrix is crucial for the organoids to maintain a clear lumen [40]. In turn, tubuloids subjected to ECM removal or digestion lose their lumen [41]. To test the impact of ECM in our system, we initially cultured freshly isolated kidney cells in low attachment plates devoid of ECM scaffold. However, the primary cells cultured in suspension did not form tubuloids and aggregated into loose clusters (Fig. S1B). However, when dissociating cultured P0 dome tubuloids into single cells and seeding them in suspension, we observed the formation of organized structures representing tubuloids (Fig. 1C, D). Interestingly, suspension tubuloids developed dense structures with smaller or no lumen over multiple passages (Fig. 1D), displayed lower proliferation rates than dome tubuloids (Fig. S1C, D), and could be maintained in culture until passage 5 (P5; around 50 days) (Fig. 1D). For this study, we successfully established tubuloid cultures from seven fresh adjacent non-neoplastic tissue from partial or complete human nephrectomy, each from a different donor, demonstrating the robustness and efficiency of our culture methods.

### Characterization of 3D in-vitro tubuloid cultures

**Fig. 2:**
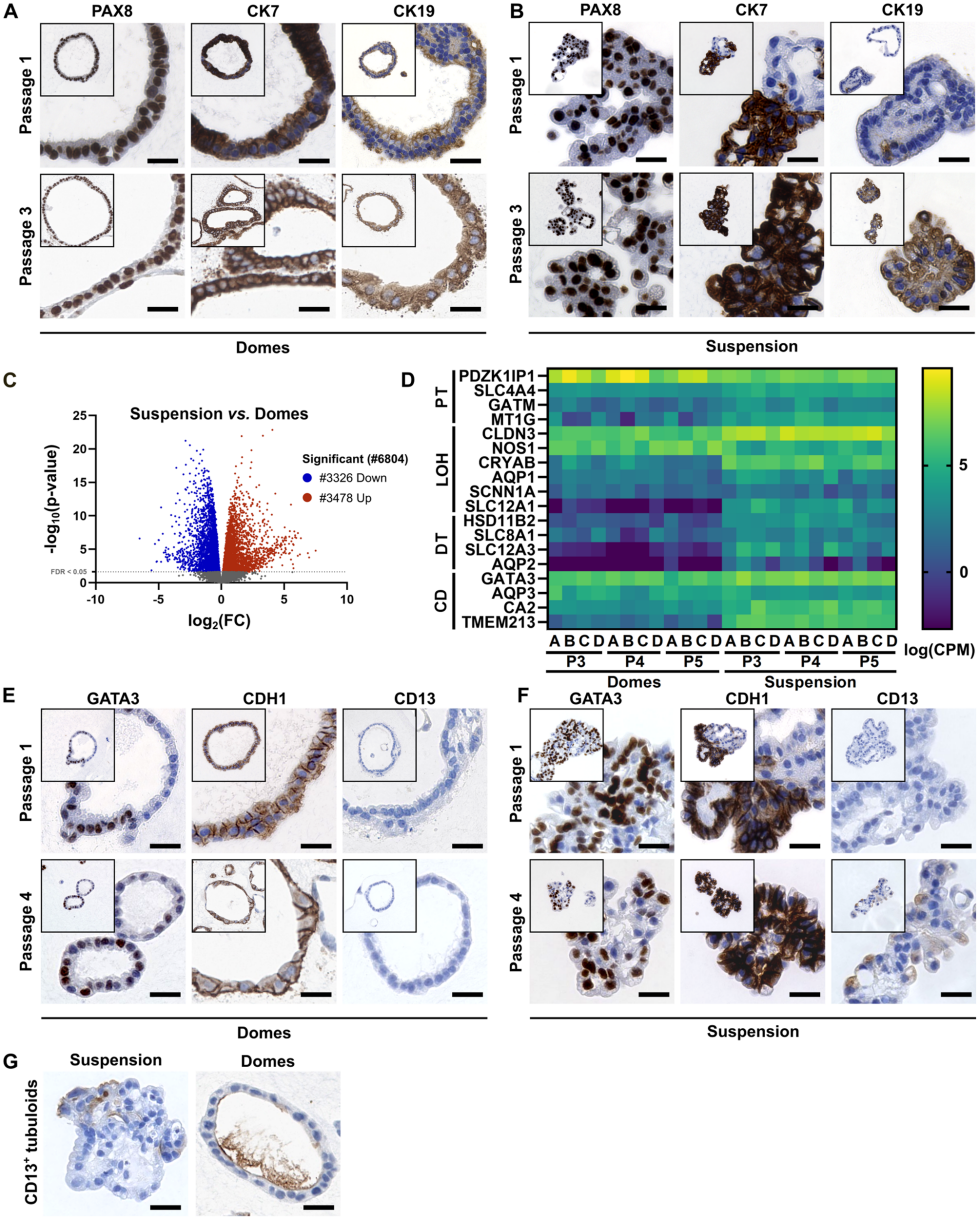
Characterization of tubuloids cultured in collagen domes and suspension. **(A, B)** Representative images of tubuloids cultured in collagen domes **(A)** or in suspension **(B)** at passages P1 or P3, labeled with PAX8, CK7, or CK19 (brown, epithelium) and stained with Haematoxylin (purple, nuclei). Scale bars: 20 μm. **(C)** Volcano plot of the 6804 differentially expressed genes between the dome and suspension tubuloids depicting their fold change (log_2_) by adjusted P-value (-log_10_). Genes with a positive fold change are defined as signature genes in suspension tubuloids, whereas those with negative fold change are in the dome tubuloids. **(D)** Gene expression heatmaps of Kidney Precision Medicine Project (KPMP) marker genes in dome and suspension tubuloids. Annotations A-D indicate different donors. The relative expression of markers is depicted as normalized counts/transcripts per million (log_CPM_). CD; collecting duct, DT; distal tubule, LOH; Loop of Henle and PT; proximal tubule **(E, F)** Representative images of tubuloids cultured in collagen domes **(E)** or in suspension **(F)** at passages P1 or P4, labeled with GATA3, CDH1, or CD13 (brown, epithelium) and stained with Haematoxylin (purple, nuclei). Scale bars: 20 μm. **(G)** Representative images of CD13^+^ tubuloids cultured in collagen domes or suspension at passage P1, labeled with CD13 (brown, epithelium) and stained with Haematoxylin (purple, nuclei). Scale bars: 20 μm

Next, we performed immunohistochemistry using different kidney-specific epithelial markers to confirm the tubular epithelial nature of the tubuloids. Regardless of the passage, all dome and suspension tubuloids were PAX8^+^ (Fig. 2A, B), demonstrating that tubuloid cultures were derived from kidney-specific epithelial cells and homogenously represented kidney epithelium. This was supported by cytokeratin 7 (CK7) and CK19 labeling, which were strongly expressed in all tubuloids at the later passages (Fig. 2A, B).

To determine the differences between dome and suspension tubuloids on a molecular level, we performed bulk RNA sequencing. We compared the transcriptome of dome and suspension tubuloids established from kidney tissue samples from four donors and cultured up to passages P3, P4, and P5. Gene expression varied significantly between dome and suspension tubuloids, irrespective of the donor and passage (Fig. S2A, B). The differentially expressed genes (DEGs) were consistent across different culture conditions (Fig. S2C-F), allowing to pool the data from different passages. Overall, 6804 genes were differentially regulated with 3478 being up- and 3326 downregulated in suspension and dome tubuloids, respectively (Fig. 2C).

We used the transcriptomic data to better characterize the cellular identity of tubuloids. To this end, we verified the expression of Kidney Precision Medicine Project (KPMP) marker genes [42] representing different parts of the kidney epithelial tubule. Markers of the endothelium (*PECAM1*, *CDH5*, *FLT1*) and interstitium/smooth muscle (*MEIS1*, *CALD1*, *PDGFRB*) were not expressed in both suspension and dome tubuloids, whereas kidney epithelial markers (*EPCAM*, *PAX8*) were highly expressed (Fig. S3A). In general, all kidney tubule-specific markers showed a higher expression in suspension compared to dome tubuloids (Fig. 2D). Notably, the expression of markers did not vary between donors and from early to late passages in both tubuloids, suggesting a stable epithelial composition. The dome and suspension tubuloids demonstrated a mixed transcriptomic expression profiles of kidney tubule-specific markers, suggesting a non-homogenous cell type composition. They expressed collecting duct (CD) markers such as *GATA3*, *AQP3*, and *CA2*, proximal tubule (PT) marker *PDZK1IP1* and the loop of Henle (LOH) marker *CLDN3* (Fig. 2D). We further validated the transcriptomic data by immunohistochemistry (Fig. 2E, F). Indeed, dome and suspension tubuloids displayed GATA3^+^ nuclei at a frequency of 50–70% (Fig. 2E, F and Fig. S3B), suggesting CD-like cells dominating but not completely constituting the tubuloids. However, most cells were CDH1^+^, a marker which is strongly expressed in distal tubule (DT) and CD, and lacked expression of the PT marker CD13, indicating a more distal cellular origin for the GATA3^−^ cell portion. In some rare cases, we detected CD13^+^ cells with some evidence of a brush border, indicating the presence of occasional PT cells in our tubuloids (Fig. 2G).

**Fig. 3:**
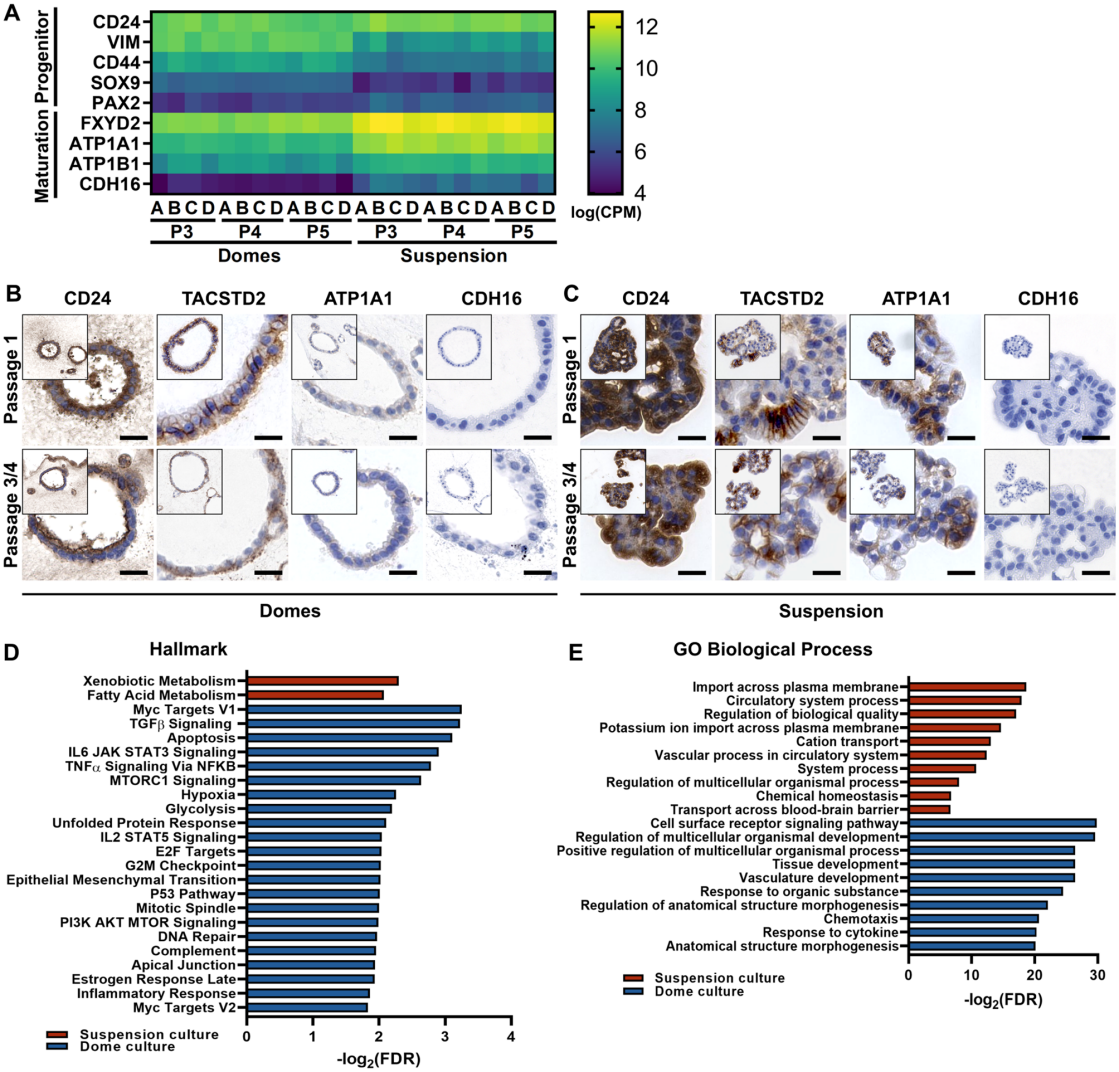
Cellular differentiation and maturation states of tubuloid cultures. **(A)** Gene expression heatmaps of differentiation and maturation markers in dome and suspension tubuloids. Annotations A-D indicate different donors. The relative expression of markers is depicted as normalized counts/transcripts per million (log_CPM_). **(B, C)** Representative images of tubuloids cultured in collagen domes **(B)** or in suspension **(C)** at passages P1 or P4, labeled with CD24, TACSTD2, ATP1A1 or CDH16 (brown, epithelium) and stained with Haematoxylin (purple, nuclei). Scale bars: 20 μm. **(D)** Enriched hallmark gene sets from MSigDB for the differentially expressed genes (suspension: red, dome: blue). The scale bar represents the adjusted False Discovery Ratio (FDR). All significantly enriched hallmark gene sets (FDR value < 0.25) with the lowest adjusted FDR (log2) of each condition were plotted. **(E)** Ontological analysis of the differently expressed genes based on GO databases (suspension: red, dome: blue). GO term enrichment was obtained from STRING network analysis. The scale bar represents the adjusted FDR. The top ten GO terms with the lowest adjusted FDR (log_2_) of each condition were plotted

As suspension tubuloids overall displayed a stronger expression of kidney-specific genes at the transcriptional level, we presumed differences in cellular maturation and differentiation states between tubuloid cultures and analyzed the transcriptomic data. Interestingly, dome tubuloids expressed dedifferentiation and regeneration markers (*CD24*, *VIM*, *CD44*, *SOX9*), whereas suspension tubuloids strongly expressed genes encoding for Na^+^/K^+^-ATPase pump (*FXYD2*, *ATP1A1*, *ATP1A2*) and other ion channels, which are markers for functional mature kidney epithelium (Fig. 3A). In agreement with the regenerative response in dome tubuloids, we also noted the absence of podocyte markers (*NPHS1*, *WT1*, *PODXL*) (Fig. S3A). Immunohistochemistry also revealed stronger expression of ATP1A1 and seemingly weaker expression of the dedifferentiation markers CD24 and TACSTD2 in suspension tubuloids, compared to dome tubuloids (Fig. 3B, C). Furthermore, we tested CDH16, also called kidney-specific (Ksp-) cadherin, which is exclusively expressed in epithelial cells of the adult kidney [43]. However, all dome and suspension tubuloids lacked CDH16 expression, independent of the passage (Fig. 3A-C).

To identify relevant pathways in dome and suspension tubuloids, we performed standard Gene set enrichment analysis (GSEA) of all Hallmark gene sets. GSEA of all expressed genes showed that tubuloids promote upregulation of genes related to tissue development, differentiation, and homeostasis (Fig. 3D). The enriched gene sets for dome tubuloids were mainly associated with cell proliferation and mitosis (Fig. 3D). For Gene Ontology (GO) analysis, the 200 signature genes of dome and suspension tubuloids with the highest fold change were plotted in a STRING network (Fig. S4). The GO Biological Process analysis revealed overexpression of genes related to ion transport across the plasma membrane and chemical homeostasis in the suspension tubuloids (Fig. 3E), in line with the higher expression of markers for mature kidney epithelia (Fig. 3A-C). In contrast, dome tubuloids displayed GO biological process profile associated with cell polarity and development (Fig. 3E). Thus, the dome tubuloids exhibit a developmental phenotype, whereas suspension tubuloids seems to be further developed, expressing markers of mature kidney epithelia.

### Chronic cAMP signalling drives cyst development in tubuloids

**Fig. 4:**
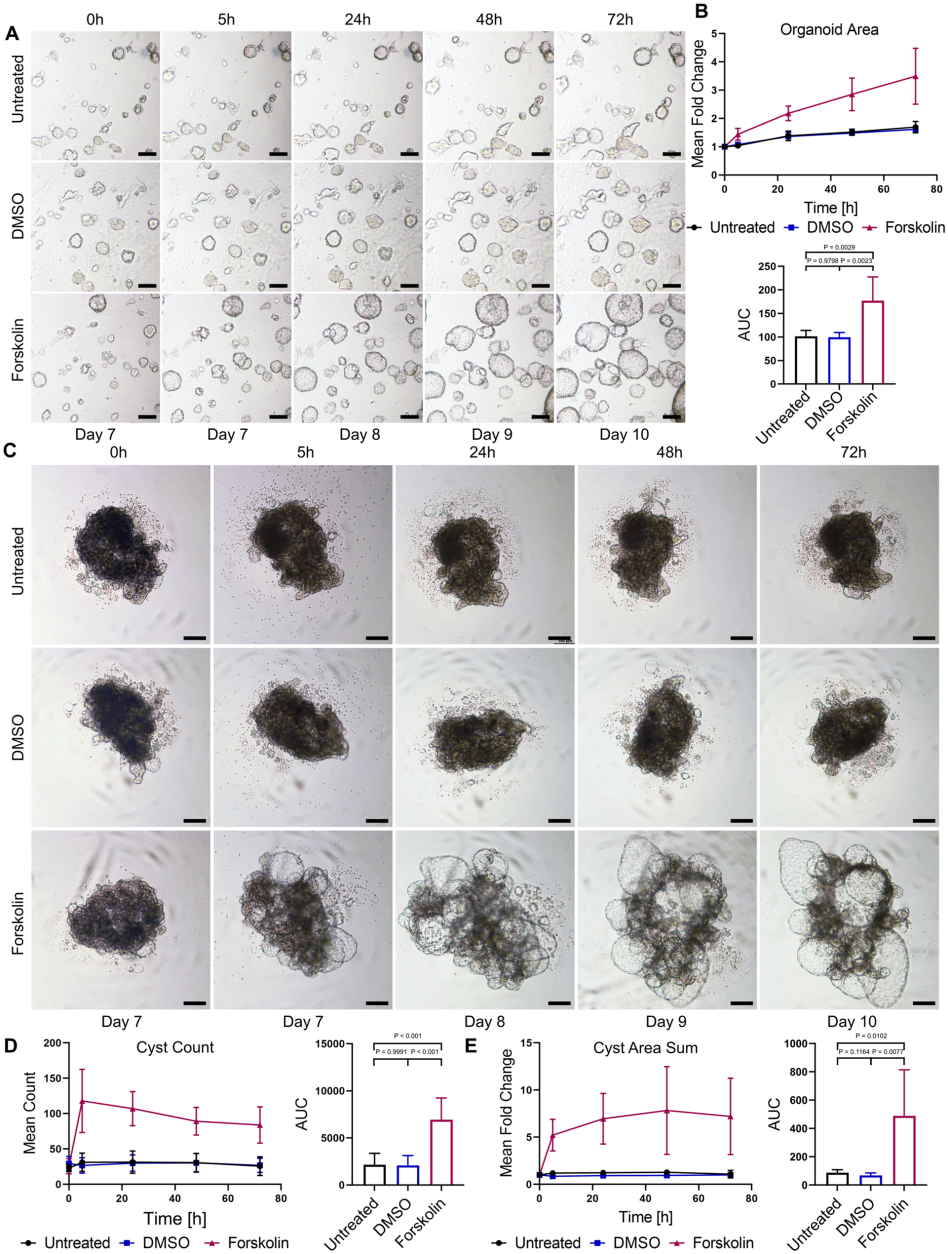
cAMP signalling drives cyst growth in dome and suspension tubuloids. **(A)** Representative bright-field images of P2 dome tubuloids cultured in medium only (untreated), DMSO, or 10 µM Forskolin supplemented medium captured 5-, 24-, 48-, and 72-hours post-treatment. Scale bars = 100 μm. **(B)** The line graph (top) shows the mean fold change of the tubuloid area. Individual data points represent the mean of independent experiments on tubuloids derived from three donors, derived from images of eight tubuloids per experiment. The bar graph (bottom) shows the areas under the curve (AUC) measured for the line graph. Data are shown as mean ± SD. P-values were calculated using a Brown-Forsythe and Welch ANOVA with Dunnett’s T3 multiple comparisons test. **(C)** Representative bright-field images of suspension tubuloids cultured in medium only (untreated), DMSO, or 10 µM Forskolin supplemented medium captured 5-, 24-, 48-, and 72-hours post-treatment. Scale bars = 100 μm. **(D, E)** The line graphs (left) show the **(D)** mean cyst count (sum) and **(E)** mean fold change of the cyst area. Individual data points represent the mean of independent experiments on tubuloids derived from three donors, derived from images of all cysts in each well per experiment. The bar graphs (right) show the AUC measured for the respective line graphs. Data are shown as mean ± SD. P-values were calculated using a Brown-Forsythe and Welch ANOVA with Dunnett’s T3 multiple comparisons test

Next, we evaluated whether dome and suspension tubuloids are applicable for studying cAMP-dependent kidney cyst formation using Forskolin, which is activates AC [44] and has been shown to induce rapid cyst formation in iPSC-derived kidney organoids [17, 18, 23, 24]. Due to their inherent cystic morphology, we did not expect Forskolin to promote the formation of additional cysts in dome tubuloids. Instead, Forskolin treatment led to an apparent swelling of dome tubuloids (Fig. 4A), which was represented by an increase in the tubuloid area compared to control conditions (Fig. 4B). This is likely due to activation of the CFTR channel, leading to Cl^−^ secretion into the lumen and, consequently, osmosis-mediated swelling of tubuloids [26, 28]. However, in suspension tubuloids, Forskolin caused cyst formation (Fig. 4C, Video S1), corroborated by the significant increase in the cyst count and area (Fig. 4D, E). Thus, our results demonstrate that suspension tubuloids develop significant and newly-formed cysts in vitro upon chronic stimulation of cAMP signalling and represent a functional tool for investigating PKD.

### Characterization of cAMP-induced cyst development in tubuloids

**Fig. 5:**
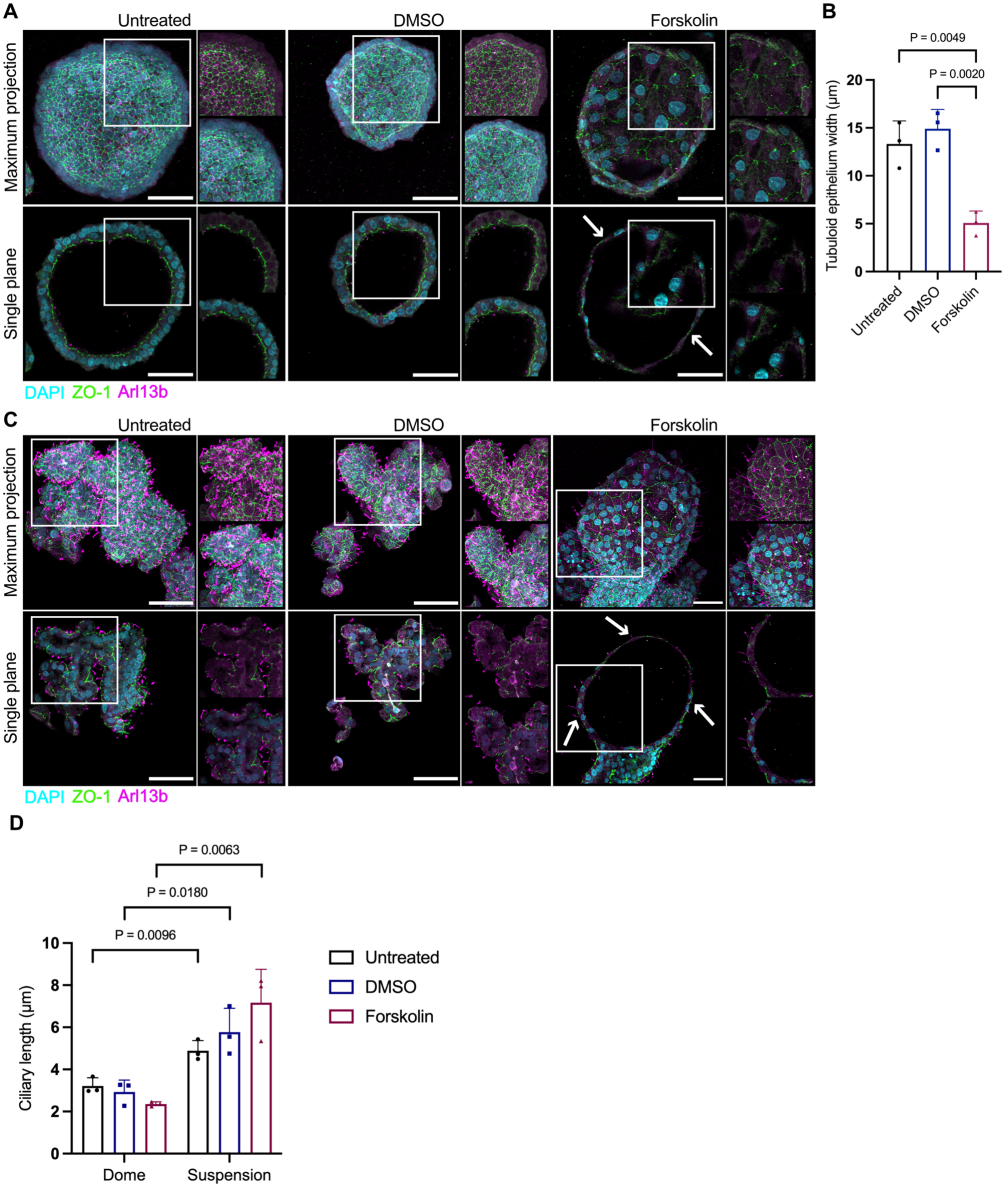
Characterization of cAMP-induced cyst growth in tubuloids. **(A)** Immunofluorescence stainings of P2 dome tubuloids cultured in medium only (untreated), DMSO, or 10 µM Forskolin supplemented medium. Tubuloids were labeled with DAPI (DNA, cyan), ARL13b (cilia, magenta), and the ZO-1 (epithelial tight junction, green). Shown are maximum intensity projections of Z-stacks through the tubuloids and single confocal slices. Arrows indicate the thinning of the tubuloid wall in Forskolin-treated dome tubuloids. Scale bars = 50 μm. **(B)** Bar graph depicting the width of dome tubuloid epithelium. Individual data points represent the mean of independent experiments on tubuloids derived from three donors, containing multiple images per experiment. Data are shown as mean ± SD. P-values were calculated using a one-way ANOVA with Tukey’s post-hoc test. **(C)** Immunofluorescence stainings of P2 suspension tubuloids cultured in organoid complete medium (untreated), DMSO, or 10 µM Forskolin supplemented organoid complete medium. Tubuloids were labeled with DAPI (DNA, cyan), the ARL13b (cilia, magenta), and the ZO-1 (epithelial tight junction, green). Shown are maximum intensity projections of Z-stacks through the tubuloids and single confocal slices. Arrows indicate the cysts in Forskolin-treated suspension tubuloids. Scale bars = 50 μm. **(D)** The bar graph shows the length of primary cilia in dome and suspension tubuloids from each experimental condition. Individual data points represent the mean of independent experiments on tubuloids derived from three donors, containing multiple images per experiment. Data are shown as mean ± SD. P-values were calculated using an unpaired, two-tailed t-test with a 95% confidence interval

To further characterize cAMP-induced cysts, we performed fluorescent immunostainings of Forskolin-treated tubuloids. We characterized the epithelial cell polarity with ARL13B (to stain for the primary cilium) and ZO-1 (to label the tight junctions of the epithelium). Immunostaining confirmed highly polarized epithelial and cystic morphology of dome tubuloids, with primary cilia facing the lumen (Fig. 5A). The basolateral membrane was directed towards the collagen-scaffold, while the apical side formed a continuous border to the fluid-filled lumen, resembling tubular morphology. Dome tubuloids retained the cystic structure after Forskolin treatment, but appeared dilated with a thinner tubuloid wall (Fig. 5A). Indeed, image analysis revealed a significant reduction in the width of tubuloid epithelium in Forskolin-treated compared to untreated dome tubuloids (Fig. 5B).

The suspension tubuloids also formed a polarized epithelium, but displayed an apical-out inverted polarity with cilia facing the outside of the tubuloid (Fig. 5C). This is in line with other reports, demonstrating the formation of polarity-inverted tubuloids in the absence of an ECM scaffold [41, 45]. Forskolin treatment promoted cyst formation with primary cilia still showing an apical-out orientation and thinning of tubuloid wall in the cystic areas (Fig. 5C). Notably, the differences in the polarized epithelial structures were recapitulated in the morphology of primary cilia in tubuloids: primary cilia in dome tubuloids were shorter compared to suspension tubuloids (Fig. 5A, C, D).

To investigate whether tubuloids are accessible to genetic approaches, which allow to manipulate signalling, we used recombinant adeno-associated virus (rAAV) transduction. We used the serotype rAAV2/8, which includes capsid proteins from serotype 8 [46] that is known to efficiently transduce kidney epithelial cells in different model systems [47]. Both dome and suspension tubuloids could be transduced with rAAV2/8-CMV-EGFP, as demonstrated by EGFP expression (Fig. S5). Thus, both tubuloids can be genetically modified, representing valuable platforms to investigate the molecular mechanisms underlying ADPKD.

## Discussion

Tubuloids have been utilized as simplified human kidney models to investigate the disease mechanisms of PKD [25, 26]. However, due to their cystic morphology, characterized by a clear central lumen surrounded by a cuboidal epithelium, kidney tubuloids are not ideal for studying kidney cystogenesis, limiting their application to model PKD. Typically, tubuloids are generated by culturing primary kidney epithelial cells encapsulated in a basement membrane gel or collagen matrix [25–28]. Extracellular cell matrix (ECM) facilitates 3D growth of tubuloids by providing spatial cues that support epithelial polarity. A recent study highlighted the role of ECM in maintaining their cystic morphology, as tubuloids subjected to ECM removal or digestion lose their lumen [41].

Here, we present a comparative study on distinct in-vitro tubuloid cultures with and without ECM scaffold and their utility in modelling kidney cystogenesis. Similar to conventional tubuloids, our dome tubuloids developed into mono-layered cystic structures. In contrast, suspension tubuloids formed self-aggregated multi-layered structures with small or no lumen. Adding on to the investigation of tubuloid structures, ZO-1, a key protein in the apical tight junctional complex, revealed distinctive localization patterns: it was localized on the inner surface of dome tubuloids and the outer surface of suspension tubuloids. These localization patterns of ZO-1 are consistent with previous literature on tubuloids [26, 28, 41] as well as in-vivo observations [48]. Dome tubuloids displayed primary cilia facing from the apical membrane into the lumen, whereas in suspension tubuloids, the cilia faced outward. Altogether, these findings indicate the specific orientations of the apical membranes, suggesting basal-out polarity in dome tubuloids and apical-out polarity in suspension tubuloids. Of note, suspension tubuloids had a surface densely packed with long primary cilia, approximately 4–6 μm in length, compared to the typical length of 1–3 μm for primary cilia in healthy adult human kidneys [49]. The elongation of cilia is likely not solely due to the polarity reversal; it may also be influenced by the absence of fluid shear stress and downstream cilium-mediated signalling [50, 51]. These aspects warrant further investigation in future studies.

There are, however, limitations to the suspension tubuloid cultures. (1) Long-term expansion of suspension tubuloids was not possible due to their low proliferation rates and mature phenotype. Overall, passaging them was impractical; instead, deriving them from dome tubuloids at the respective passages presented a much more feasible solution. Future studies may optimize specific growth factor conditions to efficiently expand suspension tubuloids. (2) Unlike conventional tubuloids, suspension tubuloids lacked a regular structure and varied in size. The transition to apical-out polarity inherently introduces some variability in both shape and structure. However, to ensure that each suspension tubuloid could achieve a relatively uniform size, a non-adherent cell culture platform like StemFIT 3D culture system with microwells could be utilized [41].

The expanding dome and suspension tubuloids contain kidney epithelial cells representing distinct nephron segments, most notably of the collecting duct (CD), but also of distal tubule (DT), proximal tubule (PT) and loop of Henle (LOH). Tubuloids demonstrated transcriptomic and protein expression profiles of kidney tubule-specific markers that aligned with the CD phenotype: (1) CD-specific transcription factor GATA binding protein 3 (GATA3) and the water channel Aquaporin 3 (*AQP3*) necessary for proper CD epithelial function were expressed. (2) DT and CD marker Cadherin-1 (CDH1), was also strongly expressed in most tubuloids. The expression profiles also matched proximal nephron (PT and LOH) patterns in Claudin-3 (*CLDN3*), PDZK1 interacting protein 1 (*PDZK1IP1*) expression, and other minor genes, though no specific markers suggested pronounced involvement of these cell types. The tubuloids, particularly dome tubuloids, displayed low expression of distal tubule-specific markers, including kidney-specific cadherin (CDH16), which is observed predominantly in the distal portion of the nephron [52]. The strong expression pattern of CK7 and CK19, as reported previously in adult kidney tissue [53], indicate the presence of collecting duct cells in tubuloids. The collecting duct has long been considered the predominant site of origin for cysts in patients with ADPKD [54]. Thus, our tubuloids, especially the suspension tubuloids, are a relevant model for studying disease mechanisms of PKD, in addition to the existing human iPSC-derived collecting duct organoids [18, 22] and collecting duct tubuloids [45].

Tubuloids comprise adult kidney tubular epithelium exhibiting a stem cell signature and regenerative response in vitro [26–28]. The expression profile of our dome tubuloids fits stemness, proliferation, and dedifferentiation, indicating that they recapitulate kidney repair. The absence of fluid flow in dome tubuloids might render the functional mechanosensitive PC1/PC2 complex inactive, alleviating the Ca^2+^-dependent inhibition of AC and subsequently activating kidney proliferation and cystogenesis [55]. Therefore, dome tubuloids serve as a complete model of kidney cysts or kidney injury repair and can be utilized to study PKD, particularly concerning cyst enlargement. Suspension tubuloids on the other hand represent functional and mature kidney tubular epithelium, as suggested by strong expression of key transporter proteins such as Na^+^/K^+^-ATPase pump. They replicate essential aspects of adult kidney function, including ion transport, thus providing a relevant model for studying kidney physiology and pathology. Interestingly, the tumor-associated Calcium Signal Transducer 2 (TACSTD2) which has been recently reported to be upregulated in kidney cysts [56], is strongly expressed in both dome and suspension tubuloids, mimicking aberrant/increased cell proliferation as observed in kidney injury repair and PKD.

PKD is associated with dysregulation of cAMP signalling in kidney epithelial cells, transforming kidney tubules into cysts through increased proliferation and fluid secretion. Previous studies have shown that treatment with Forskolin can enlarge cystic structures in tubuloids [25, 26]. We observed similar cyst dilation in dome tubuloids following chronic cAMP stimulation, which we validated by significantly reduced tubuloid wall thickness and a loss of epithelial barrier integrity. In response to chronic cAMP signalling stimulation, suspension tubuloids exhibit rapid and significant cystogenesis with newly-formed cysts having an apical-out epithelium. Interestingly, this is similar to what has been reported previously for *PKD1* tubuloids or *PKD* hPSC-derived kidney organoids [19, 26]. In addition, we observed significant epithelial thinning around suspension tubuloid cysts, which to our knowledge, no prior studies have reported.

Suspension tubuloid cysts exhibited fluctuations in size by expanding and constricting over time (see Video S1). This phenomenon has previously been described in cysts from PKD hPSC-derived kidney organoids as “breath-like” movements, which result from the rupture of the organoid epithelium due to expansive fluid pressure [17]. Future studies are necessary to illustrate the detailed underlying mechanism of spontaneous cyst formation in suspension tubuloids. While cAMP-mediated mTOR activation and subsequent increased proliferation are potential driving mechanisms, they are unlikely to be the only factors at play, as these tubuloids can form cysts within just 3 to 5 h post-treatment. Fluid secretion is considered to play an important role in PKD, and previous studies have suggested that cyst growth may be caused by increased secretory (basolateral-to-apical) solute transport [57–60]. In this context, it is important to further analyze the localization of ion transporters, such as CFTR and Na^+^/K^+^-ATPase, which are typically found in the apical and basolateral membrane of epithelial cells, respectively to maintain ion gradients. Given their altered polarity, it is presumed that there is a reorientation of the localization of these transporters in suspension tubuloids; however, this needs to be confirmed. Nonetheless, we demonstrate suspension tubuloids as valuable platforms to model cAMP-induced kidney cystogenesis.

Both dome and suspension tubuloids offer an additional advantage: they can be genetically manipulated, as shown in previous studies on tubuloids [26, 28]. This will allow the use of ciliary-localized optogenetic tools to specifically manipulate ciliary cAMP levels [61] in tubuloids. This approach will enhance our understanding of how ciliary cAMP signalling contributes to the development of kidney cysts in humans.

## Conclusion

Taken together, we have established versatile tubuloid culture systems for primary human kidney epithelial cells to investigate the pathogenetic mechanisms underlying ADPKD. Our study highlights apical-out suspension tubuloids as a simplified yet promising in-vitro system that allows molecular and cellular analyses and demonstrates the validity and applicability to model kidney cystogenesis. Together with conventional tubuloids, suspension tubuloids offer valuable platforms to assess the specificity and the mode of action of small molecules being developed for ADPKD treatment in physiologically relevant, tissue-like microenvironments. This will lay the groundwork for identifying novel targets to create more effective, targeted therapies for ADPKD.

## Supplementary Information

Below is the link to the electronic supplementary material.

Supplementary Material 1

Supplementary Material 2

Supplementary Material 3

Supplementary Material 4

Supplementary Material 5

Supplementary Material 6

Supplementary Material 7

Supplementary Material 8

Supplementary Material 9

Supplementary Material 10

Supplementary Material 11

Supplementary Material 12

Supplementary Material 13

## Data Availability

Non-commercially available reagents can be made available under a material transfer agreement with the University Hospital Bonn (UKB). All data inquiries should be addressed to the corresponding authors. The image datasets generated and/or analyzed during the current study are available in the bonndata repository: [https://bonndata.uni-bonn.de/previewurl.xhtml?token=8fd7ee9c-727d-4814-8737-0398942cf55d]. Bulk RNA-seq data has been deposited at the European Genome Phenome Archive under EGAD50000002335 [https://ega-archive.org/datasets/EGAD50000002335]. All other relevant data and resources can be found in the article and its supplementary information.

## Abbreviations

3D: Three-dimensional
AC: Adenylyl cyclase
ADPKD: Autosomal dominant polycystic kidney disease
ANOVA: Analysis of variance
AQP3: Aquaporin 3
ARL13b: ADP-ribosylation factor-like protein 13B
ASC: Adult stem cell
ATP1A1: ATPase, Na^+^/K^+^ transporting, alpha 1 polypeptide
AUC: Area under the curve
CA2: Carbonic anhydrase 2
cAMP: Cyclic adenosine monophosphate
ccRCC: Clear cell renal cell carcinoma
CD13: Cluster of Differentiation 13
CD24: Cluster of Differentiation 24
CDH1: Cadherin-1
CD: Collecting duct
CDH16: Cadherin-16
CFTR: Cystic Fibrosis Transmembrane Conductance Regulator
CMV: Cytomegalovirus
CPM: Counts/transcripts per million
CREB: Cyclic AMP-Responsive Element Binding-Protein
CK7: Cytokeratin 7
CK19: Cytokeratin 19
CLDN3: Claudin-3
DAPI: 4′,6-diamidino-2-phenylindole
DEG: Differentially expressed genes
DMSO: Dimethyl sulfoxide
DT: Distal tubule
EC: Endothelial cells
ECM: Extracellular matrix
EGFP: Enhanced green fluorescent protein
EpC: Epithelial cells
FC: Fold change
FDA: Food and Drug Administration
FDR: False Discovery Ratio
GATA3: GATA binding protein 3
GATM: Glycine amidinotransferase
GSEA: Gene Set Enrichment Analysis
GO: Gene Ontology
H&E: Haematoxylin & Eosin
HEK-293T: Human Embryonic Kidney 293T
hPSC: Human pluripotent stem cell
I/SMc: Interstitium/smooth muscle cells
iPSC: Induced pluripotent stem cells
KPMP: Kidney Precision Medicine Project
Ksp: Kidney-specific
LOH: Loop of Henle
MT1G: Metallothionein 1G
mTOR: Mammalian target of rapamycin
N: Noggin
P: Passage
PAX8: Paired box 8
PC1: Polycystin-1
PC2: Polycystin-2
PCA: Principal Component Analysis
pCREB: Phosphorylated CREB
PDZK1IP1: PDZK1 interacting protein 1
PKA: Protein Kinase A
PKD: Polycystic Kidney Disease
Pod: Podocytes
PT: Proximal tubule
rAAV: Recombinant adeno-associated viruses
RCC: Renal cell carcinoma
RNA-seq: RNA sequencing
R: R-spondin 3
SD: Standard deviation
STRING: Search Tool for the Retrieval of Interacting Genes/Proteins
TACSTD2: Tumor-associated calcium signal transducer 2
ULA: Ultra-low attachment
ZO-1: Zonula Occludens-1
µm: Micrometres
µM: Micromolar
W: Wnt-3a
