# Supplementary figures and images for: Modelling kidney cystogenesis using human kidney tubuloid cultures
Source: BMC Mol Cell Biol. 2026 May 11;27:26. doi: 10.1186/s12860-026-00591-x (PMC13162504; doi:10.1186/s12860-026-00591-x)

**Fig. S1**

**A**

**P6**

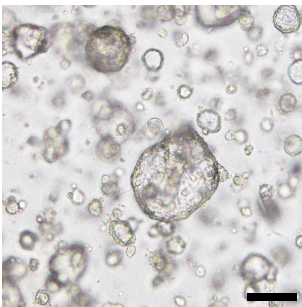

**P7**

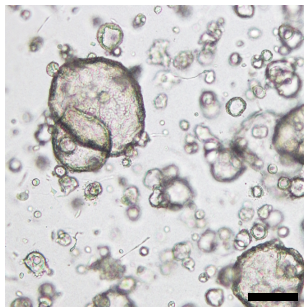

**P8**

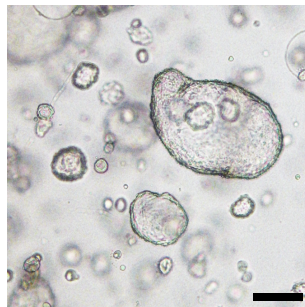

**B**

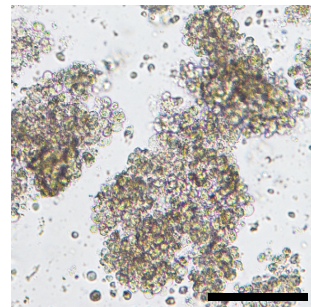

**C**

**dome culture**

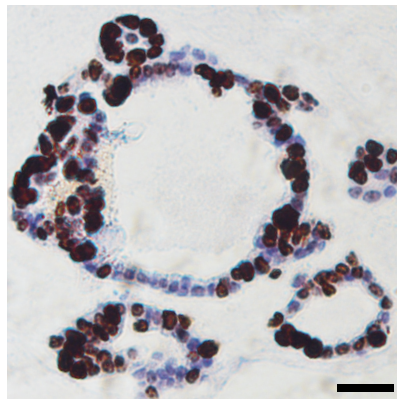

**suspension culture**

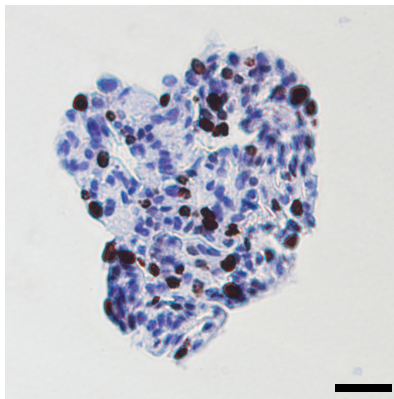

**D**

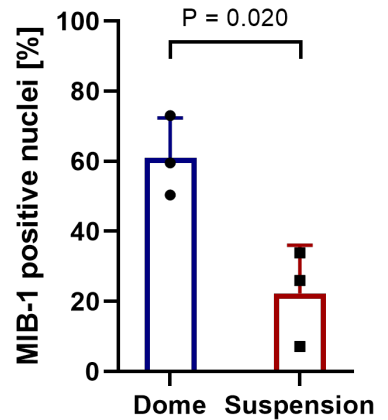

Supplement: Supplementary file 1 — Supplementary Material 1 [file 12860_2026_591_MOESM1_ESM.pdf]

**Fig. S2**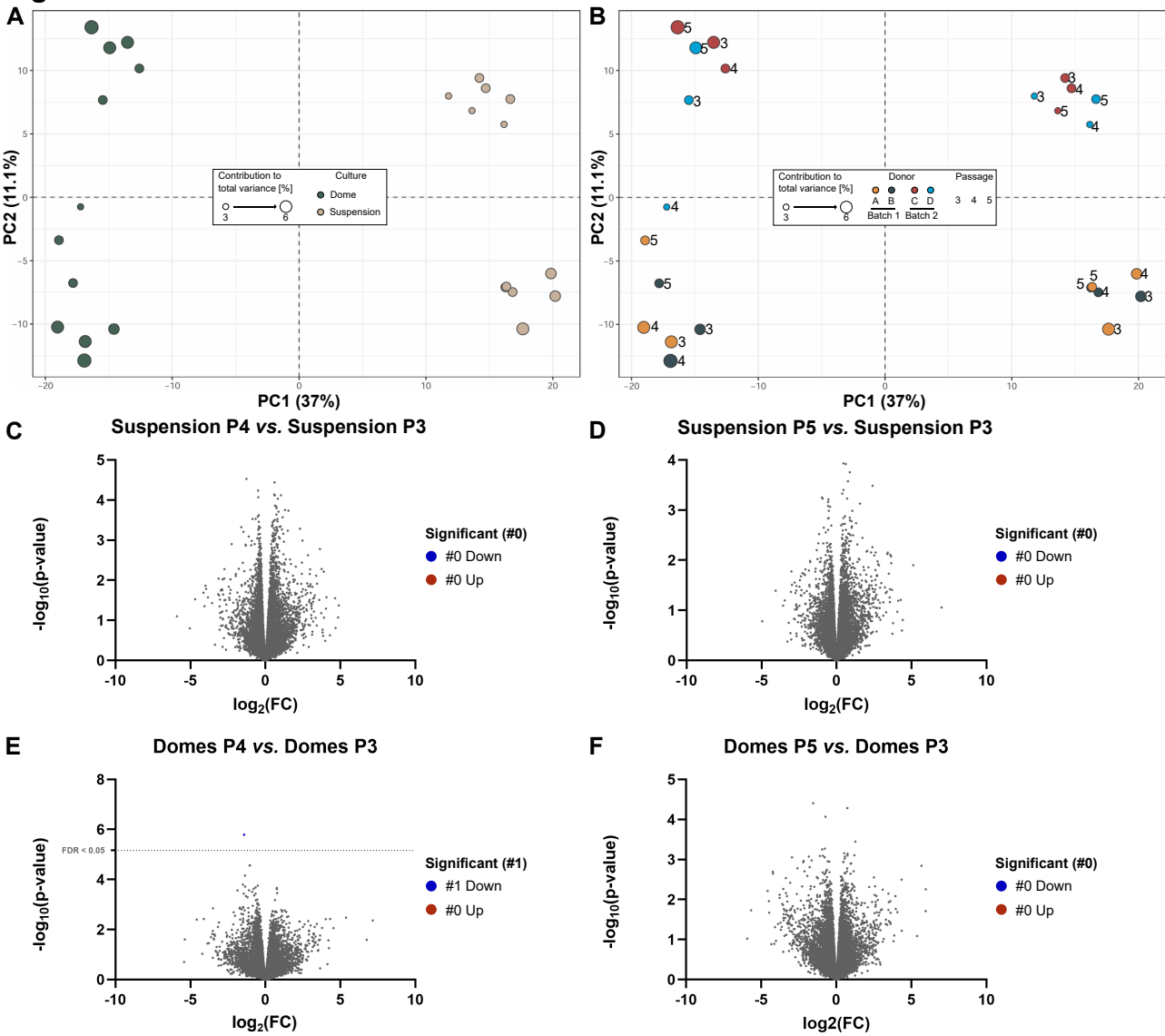

Supplement: Supplementary file 2 — Supplementary Material 2 [file 12860_2026_591_MOESM2_ESM.pdf]

**Fig. S3****A**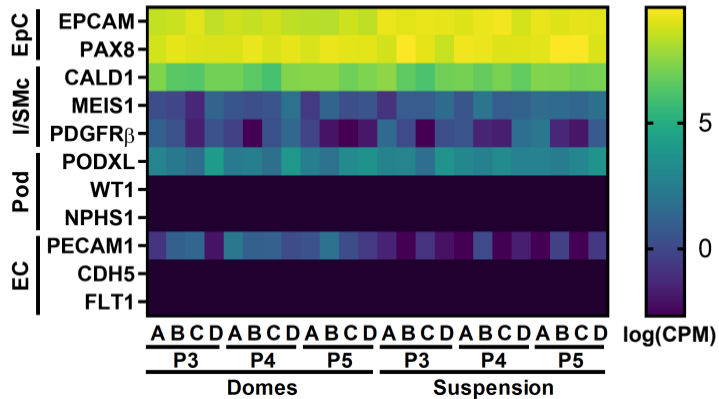**B**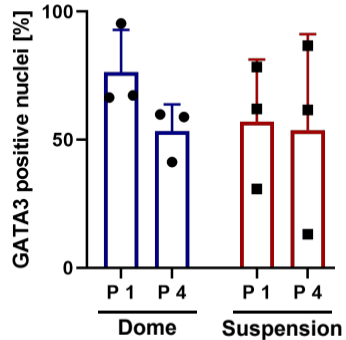

Supplement: Supplementary file 3 — Supplementary Material 3 [file 12860_2026_591_MOESM3_ESM.pdf]

# A

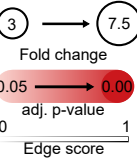

**B**

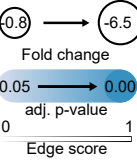

Supplement: Supplementary file 4 — Supplementary Material 4 [file 12860_2026_591_MOESM4_ESM.pdf]

**Fig. S5**

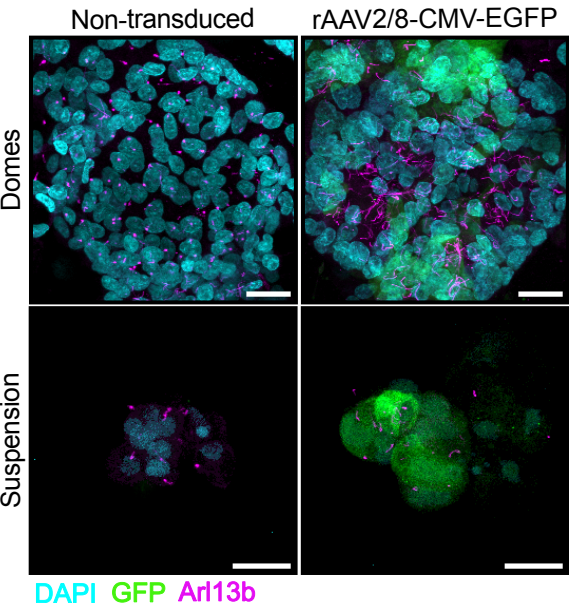

Supplement: Supplementary file 5 — Supplementary Material 5 [file 12860_2026_591_MOESM5_ESM.pdf]
