# Supplementary material for: Modelling kidney cystogenesis using human kidney tubuloid cultures
Source: BMC Mol Cell Biol. 2026 May 11;27:26. doi: 10.1186/s12860-026-00591-x (PMC13162504; doi:10.1186/s12860-026-00591-x)
Supplement: Supplementary file 13 — Supplementary Material 13 [file 12860_2026_591_MOESM13_ESM.pdf]

Supplementary information

Supplementary figures

Fig. S1

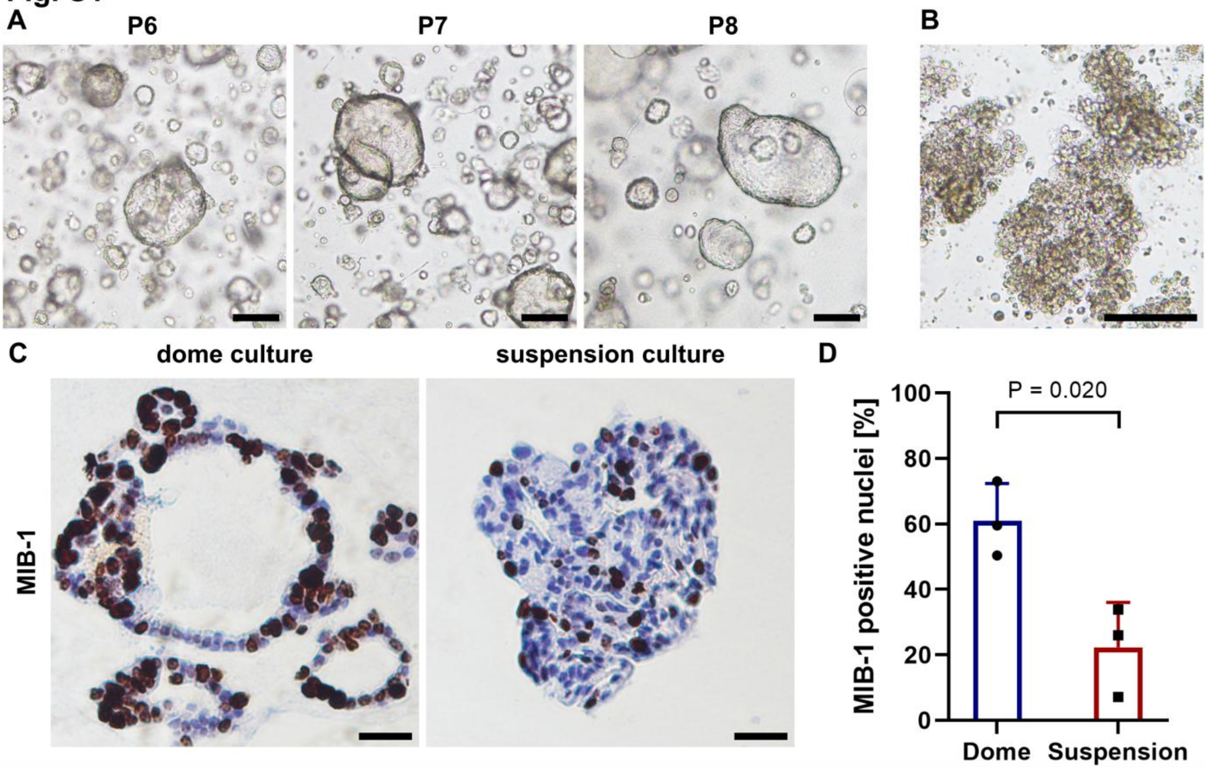

Proliferation rates in dome and suspension tubuloids. (A) Representative bright-field images of dome tubuloids cultured in collagen domes at different passages (P), from P6 to P8. Scale bars: 100 µm. **(B)** Representative bright-field image of aggregated loose clusters of cells isolated from fresh adult kidney and cultured in suspension. Scale bar: 100 µm. **(C)** Representative images of dome and suspension tubuloids at passage P1, labeled with MIB-1 (brown, nuclei) and stained with Haematoxylin (purple, nuclei). Scale bars: 20 µm. **(D)** The bar graph shows the relative proliferation rate represented as percentage of $MIB1^+$ nuclei in dome and suspension tubuloids at passage P1. Individual data points represent the mean of independent experiments on tubuloids derived from three donors, derived from images of multiple tubuloids per experiment. Data are shown as mean ± SD. P-values were calculated using an unpaired, two-tailed t-test with a 95% confidence interval.

Fig. S2

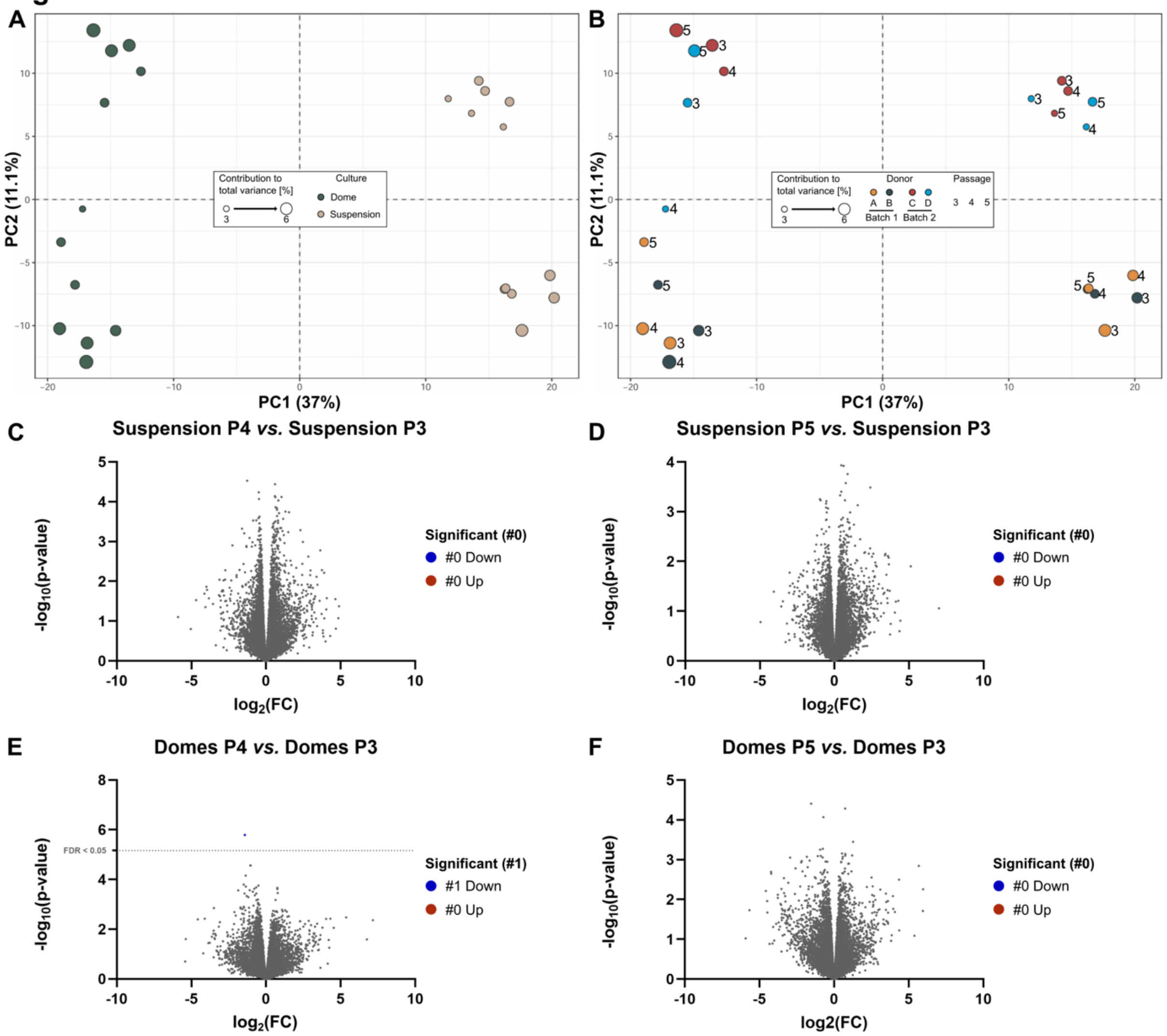

Fig. S2: Bulk RNA-Seq dataset processing. (A, B) Principal component analysis (PCA) of gene expression variation between the dome and suspension tubuloids derived from four donors and harvested at passages P3, P4, and P5. Each dot represents one single sample. The size of the dot indicates its contribution to the total variance. PCA plots for (A) sequenced samples representing dome (green) and suspension tubuloids (beige) and, (B) donors (marked by color), passages (annotated next to each sample), and batch identities. **(C-F)** Volcano plots of the differentially expressed genes between the suspension tubuloids at different passages (C) P4 and P3, (D) P5 and P3 and dome tubuloids at different passages (E) P4 and P3, (F) P5 and P3 depicting their fold change (log_2) by adjusted P-value ($-log_{10}$). Positive fold change values are signature genes in the higher passage tubuloids, while negative ones are in the lower passage tubuloids.

Fig. S3

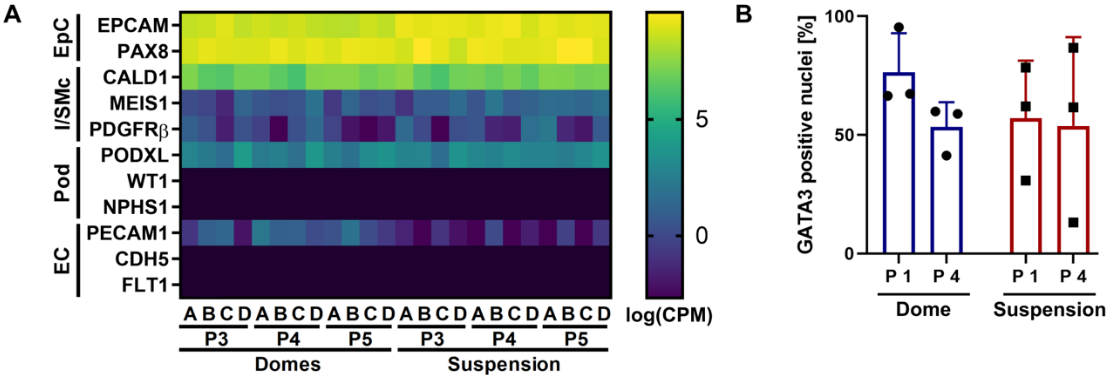

Gene expression profile in dome and suspension tubuloids. (A) Gene expression heatmaps of marker genes for endothelium, podocytes, interstitium/smooth muscle, and kidney epithelium in dome and suspension tubuloids. Annotations A-D indicate different donors. The relative expression of markers is depicted as normalized counts/transcripts per million ($\log_{CPM}$). EC; endothelial cells, Pod; podocytes, I/SMc; interstitium/smooth muscle cells and EpC; epithelial cells **(B)** The bar graph shows the percentage of GATA3^{+} nuclei in dome and suspension tubuloids at passages P1 or P4. Individual data points represent the mean of independent experiments on tubuloids derived from three donors, derived from images of multiple tubuloids per experiment. Data are shown as mean ± SD.

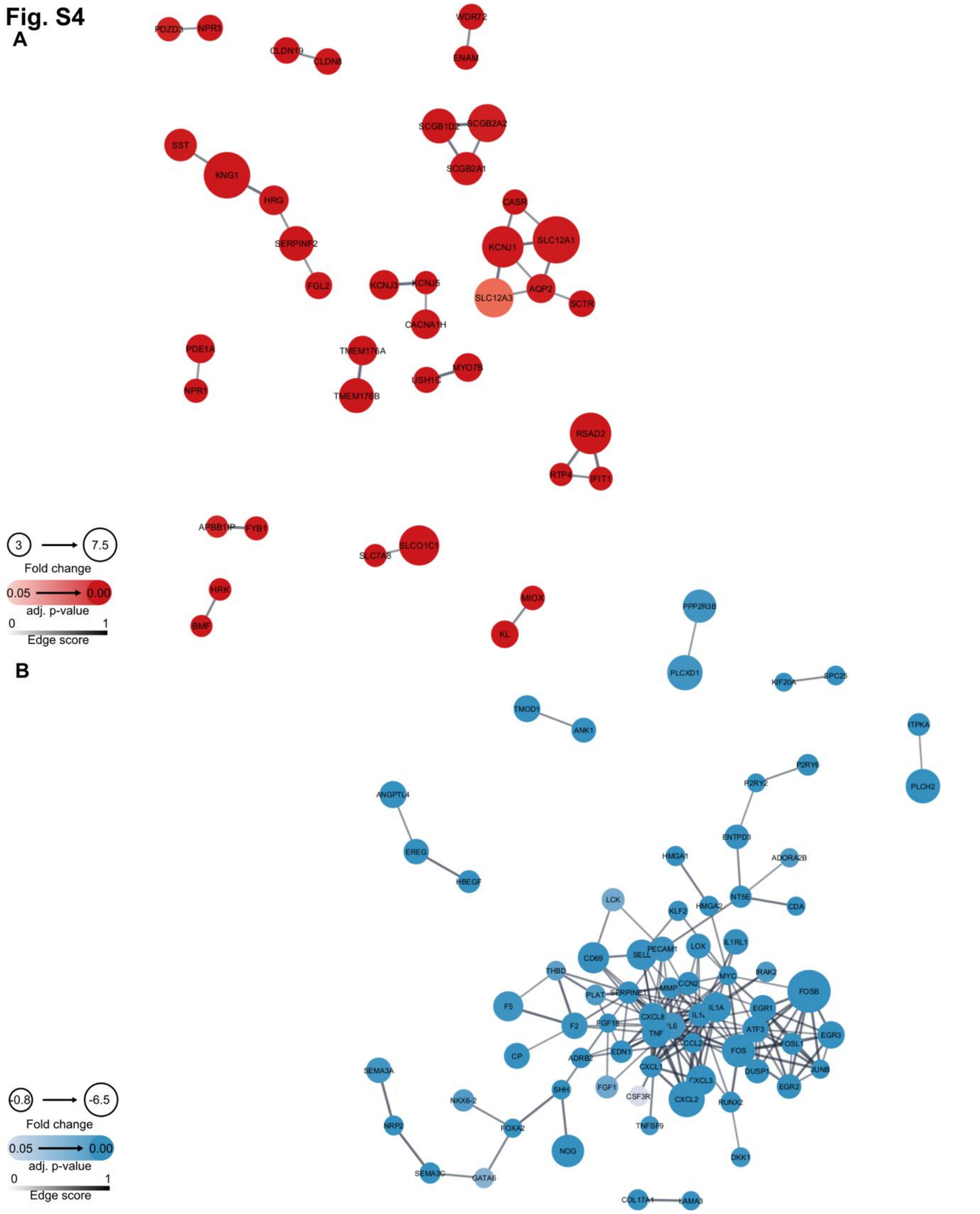

Fig. S4: STRING network analysis for differential gene expression in dome and suspension tubuloids. STRING networks of the top 200 significantly differently expressed genes in (A) suspension and (B) dome tubuloids. Nodes represent signature genes, and edges represent protein-protein associations based on known and predicted interactions, as well as text mining, co-expression, and protein homology. The edge score describes the strength of node interactions and is indicated by the edge opacity. Node sizes visualize the fold change of the corresponding gene's expression, and the node color indicates the adjusted P-value.

Fig. S5

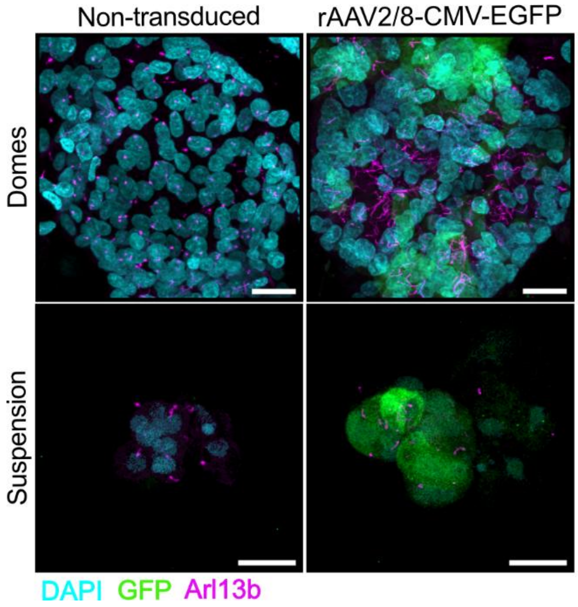

Fig. S5: Transduction of dome and suspension tubuloids. Immunofluorescence stainings of non- and rAAV2/8-CMV-EGFP-transduced P3 dome and suspension tubuloids. Tubuloids were labeled with DAPI (DNA, cyan), ARL13b (cilia, magenta), and GFP (transduction efficiency, green). Shown are maximum intensity projections of Z-stacks through the tubuloids. Scale bars = 20 μm.

Supplementary video

Video S1

Live-cell analysis of cyst formation in suspension tubuloids. Representative phase-contrast and fluorescent images of P2 suspension tubuloids stained with Calcein-AM (green) and cultured in medium only (untreated), DMSO, or 10 μM Forskolin supplemented medium. Images were captured every three hours, and treatment started after four days of culture. Scale bars = 500 μm.

Supplementary tables

Tab. S1: MIB-1 quantification (submitted as excel file)

Tab. S2: GATA3 quantification (submitted as excel file)

Tab. S3: STRING GO-Biological Process Average Suspension versus Dome (submitted as excel file)

Tab. S4: GSEA Hallmark Details (submitted as excel file)

Tab. S5: GSEA WikiPathways Details (submitted as excel file)

Tab. S6: Suspension tubuloid total cyst count (submitted as excel file)

Tab. S7: Immunohistochemistry antibodies

Specificity	Clone	Dilution	Manufacturer	Catalogue number
Ksp-Cadherin (CDH16)	EPR13090	1:1000	Abcam	#ab183745
CK7	OV-TL12/30	1:100	Agilent	#M7018
CK19	RCK108	1:1000	Agilent	#M0888
Ki-67	MIB-1	1:100	Agilent	#M7240
PAX8	MRQ-50	1:100	Cell Marque	#363M-16
GATA3	L50-823	1:50	BioCare	#CM405B
CDH1	EP700Y	1:50	Cell Marque	#246R-16
CD13	2D8D11	1:5000	Proteintech	#66211-1-Ig
TACSTD2	01	1:1500	Enzo	#ENZ-ABS380-100
ATP1A1	EP1845Y	1:4000	Abcam	#ab76020
CD24	serum	1:25	Kindly supplied by the routine laboratory of the institute of pathology, University Hospital Bonn (UKB)	-

Tab. S8: Immunofluorescence antibodies

Reactivity	Species	Fluorochrome	Dilution	Manufacturer	Catalogue number
GFP	Rabbit	NA	1:500	Abcam	#ab6556
RFP	Rabbit	NA	1:500	Rockland Immunochemicals Inc.	#600-401-379
ARL13B	Mouse	NA	1:500	Abcam,	#ab136648
ZO-1	Mouse	NA	1:250	Invitrogen	#33-9100
Rabbit	Donkey	Alexa Fluor 647	1:500	Dianova	#711-605-152
Mouse	Donkey	Alexa Fluor 647	1:400	Jackson ImmunoResearch	#715-545-150
Mouse-IgG2a	Goat	Alexa Fluor 647	1:1000	Invitrogen	#A21241
Rabbit	Goat	Alexa Fluor 647	1:500	Invitrogen	#A11034

Tab. S9: Basic organoid medium recipe, 500 ml (2x)

Volume /Amount	Product	Manufacturer	Catalogue Number	Final Concentration
440 ml	Gibco®Advanced DMEM F12	Gibco	#12634028	
20 ml	B27 supplement, minus Vitamin A, 50x	Thermo Fisher Scientific Inc.	#12587001	2x (1:25)
10 ml	Gibco® Pen Strep	Thermo Fisher Scientific, Inc.	#15140122	2x (1:50)
10 ml	Gibco® GlutaMAX	Thermo Fisher Scientific, Inc.	#35050061	2x (1:50)
10 ml	Roti®-CELL HEPES Solution	Carl Roth	#9157.1	2x (20 mM)
10 ml	N-2 Supplement, 100x	Thermo Fisher Scientific, Inc.	#17502001	2x (1:50)
203 mg	N-Acetyl-L-Cystein	Acros Organics	#160280250	2x (2.5 mM)
1.220 g	Nicotinamide	Sigma-Aldrich	#N0636	2x (20 mM)
20 µl	Gastrin-1, human Leu15 (1 mg/ml)	Eurogentec	#AS-64149	2x (10 nM)

Tab. S10: Organoid complete medium recipe, 50 ml

Volume	Product	Manufacturer	Catalogue Number	Final Concentration
25 ml	Basic organoid medium	-	-	1x
25 ml	L-WRN conditioned medium	-	-	1x
100 µl	Normocin™ (50 mg/ml)	InvivoGen	#ANT-NR-1	100 µg/ml
32 µl	Y-27632 dihydrochloride (5 mg/ml)	Biomol GmbH	#AG-CR1-3564-M010	10 µM
25 µl	EGF, recombinant, human (100 µg/ml)	Sigma-Aldrich	#SRP3027	50 ng/ml
2 µl	SB 202190 (10 mg/ml)	BioGems International Inc.	#1523077	1.2 µM
2 µl	A83-01 (5 mg/ml)	Sigma-Aldrich	#SML0788	0.5 µM

Tab. S11: Collection medium recipe, 50 ml

Volume	Product	Manufacturer	Catalogue Number	Final Concentration
48.4 ml	Gibco®Advanced DMEM F12	Gibco	#12634028	
500 µl	Gibco® GlutaMAX	Thermo Fisher Scientific, Inc.	#35050061	1x (1:100)
500 µl	Roti®-CELL HEPES Solution	Carl Roth	#9157.1	1x (10 mM)
500 µl	Amphotericin B (250 mg/ml)	Thermo Fisher Scientific, Inc.	#15290018	2.5 µg/ml
100 µl	Normocin™ (50 mg/ml)	InvivoGen	#ANT-NR-1	100 µg/ml

Tab. S12: Ham's F12 buffer recipe, 10 ml (10x)

Volume /Amount	Product	Manufacturer	Catalogue Number	Final Concentration
10 ml	ddH_20	-	-	-
1.0626 g	Gibco® Ham's F12 Nutrient Mix, powder	Thermo Fisher Scientific, Inc.	#21700018	10x (106.26 g/L)
117.6 mg	$NaHCO_3$ (Sodium bicarbonate)	Carl Roth	#0965.1	10x (11.76 g/L)

Tab. S13: Reconstitution buffer recipe, 50 ml (10x)

Volume /Amount	Product	Manufacturer	Catalogue Number	Final Concentration
38 ml	ddH_20	-	-	-
10 ml	Roti®-CELL HEPES Solution	Carl Roth	#9157.1	200 mM
2 ml	NaOH, (Sodium hydroxide, 1 N, Titripur® Reag. Ph Eur,Reag. USP)	Merck KGaA	#1091371000	50 mM
1.1 g	$NaHCO_3$ (Sodium bicarbonate)	Carl Roth	#0965.1	22 mg/ml
